# Biodegradation of S-Triazine Herbicides Under Saline Conditions by *Paenarthrobacter ureafaciens* PC, a New Halotolerant Bacterial Isolate: Insights into Both the Degradative Pathway and Mechanisms of Tolerance to High Salt Concentrations

**DOI:** 10.3390/microorganisms13030649

**Published:** 2025-03-12

**Authors:** Chunqing Fu, Yifan Jiang, Bingwen Xu, Xinmei Fu, Liang Tan, Mei Jin

**Affiliations:** 1 School of Life Science, Liaoning Normal University, Dalian 116081, China; 2 Dalian Center for Certification and Food and Drug Control, Technology Innovation Center of Rapid Screening and Traceability for Edible Agricultural Product Safety, State Administration for Market Regulation, Dalian 116037, China; 3 School of Chemical Engineering, Dalian University of Technology, Dalian 116024, China

**Keywords:** s-triazine herbicides, saline environments, *Paenarthrobacter ureafaciens* PC, biodegradation pathway, halotolerance mechanism

## Abstract

In this study, a halotolerant bacterial strain was isolated and identified. This bacterium was confirmed to efficiently degrade s-triazine herbicides under saline conditions. The optimal conditions for the metabolism and growth of this strain were determined through single-factor tests. Furthermore, the biodegradation pathways of prometryne (the target compound) by this strain were proposed based on the detection of possible degradation intermediates and genome sequencing analysis. Additionally, a possible halotolerance mechanisms of this strain were also revealed through screening halotolerance-related genes in its genome. The results demonstrated that a halotolerant bacterial strain (designated PC), which completely degraded 20.00 mg/L prometryne within 12 h under saline conditions (30.0 g/L NaCl), was isolated and identified as *Paenarthrobacter ureafaciens*. The optimal conditions for the metabolism and growth of the strain PC were identified as follows: yeast extract as the additional carbon source with the concentration of ≥0.1 g/L, NaCl concentration of ≤30.0 g/L, initial pH of 7.0, temperature of 35.0 °C, and shaking speed of ≥160 rpm. Furthermore, the strain PC demonstrated efficient removal of other s-triazine herbicides, including atrazine, ametryne, simetryne, and cyanazine. The strain PC might degrade prometryne through a series of steps, including demethylthiolation, deisopropylamination, deamination, dealkalation, decarboxylation, etc., relying on the relevant functional genes involved in the degradation of s-triazine compounds. Furthermore, the strain PC might tolerate high salinity through the excessive uptake of K^+^ into cells, intracellular accumulation of compatible solutes, and production of halophilic enzymes. This study is expected to provide a potentially effective halotolerant bacterium for purifying s-triazine pollutants in saline environments.

## 1. Introduction

S-triazine herbicides are the most widely used pesticides in the world and can inhibit the growth of broadleaf weeds and annual grasses by interfering with the normal function of photosynthesis [1]. However, the residues of s-triazine herbicides are unavoidable during application [2]. It has been reported that approximately 70% of unused s-triazine herbicides enter the aquatic ecosystem through leaching, subsurface runoff, and surface runoff [3], causing serious damage to the aquatic environment and organisms. Furthermore, the uptake of some s-triazine herbicides (e.g., prometryne) by fruit, vegetables, and cereal crops can lead to their accumulation in humans through the biomagnification effect, even threatening human health and ecological security [4,5]. Therefore, the residues of s-triazine herbicides in the environment must be converted into less toxic products or completely mineralized.

Many physical and chemical treatment technologies, such as adsorption, oxidation, photocatalysis, membrane separation, electro-Fenton, etc., have been applied to remove residual s-triazine herbicides from various polluted environments [6,7]. However, disadvantages such as severe reaction conditions, high operating costs, and potential production of secondary pollutants limit their widespread use [6]. By contrast, biological technologies attract a great deal of attention due to their advantages, such as cost-effectiveness, high treatment efficiency, and environmental friendliness [6], and thus are widely applied for the treatment of various environmental pollutants. As the most important factor in biotreatment, various microorganisms have been reported to effectively degrade s-triazine herbicides [8]. In recent years, many studies have exploited many microbial isolates capable of efficiently and stably degrading s-triazine herbicides [9]. For instance, *Pseudomonas stutzeri* Y2 was confirmed to completely degrade 50 mg/L simazine within 4 d [10]. Another bacterial strain, *Arthrobacter* sp. C2, was also confirmed to degrade 81.36% of 100 mg/L atrazine within 24 h [11]. In recent decades, there have been an increasing number of reports on the biodegradation of other s-triazine herbicides (e.g., prometryne) in addition to atrazine by microbial strains, such as *Arthrobacter aurescens* TC1 [12], *Leucobacter triazinivorans* JW-1 [13], and *Nocardioides* sp. DN36 [14]. In general, s-triazine herbicides are first converted to the corresponding hydroxyl analogues by microorganisms catalyzed by hydrolases such as triazine hydrolase (TrzN) or atrazine chlorohydrolase (AtzA) [15]. In addition, some other hydrolases have been found to catalyze the first biodegradation step of s-triazine compounds such as prometryne [4]. The corresponding hydrolysates are then usually converted to cyanuric acid by amido hydrolases, such as hydroxydechloroatrazine ethylaminohydrolase (AtzB) and N-isopropylammelide isopropylaminohydrolase (AtzC) [16]. Cyanuric acid is then further degraded and finally mineralized to ammonia and carbon dioxide by cyanuric acid amidohydrolase (AtzD), 1-carboxybiuret hydrolase (AtzE), and allophanate hydrolase (AtzF) [17]. Although many microbial strains capable of degrading s-triazine herbicides have been isolated and studied, more species with higher degradation efficiency and greater environmental adaptability should be exploited to meet the practical applications.

As reported, s-triazine herbicides are not only found in low-salt environments, such as soil and freshwater [18], but also in saline environments, such as oceans [19]. For instance, a total of ten kinds of s-triazine herbicides were detected in the sediments of Laizhou Bay in China, with concentrations ranging from 0.14 μg/kg to 1.68 μg/kg. Among them, prometryne was found to be the most prevalent, accounting for 63.50% of the total detected [19]. Meanwhile, Liu et al. [20] indicated that wastewater from many pesticide production processes also contained a high load of salts. Saline wastewaters are generally defined as the effluents containing at least 3% (*w*/*v*) salinity, as well as other numerous harmful organic and inorganic pollutants, such as heavy metals, aromatic amines, and pesticides [21]. In the context of conventional bioprocesses (e.g., activated sludge), the microorganism under investigation is generally non-halotolerant. Consequently, the presence of a high concentration of salt will result in the inhibition of the microorganism, thereby significantly decreasing the treatment efficiency of saline wastewaters [22]. In contrast, it has been demonstrated that certain microorganisms are capable of growing and maintaining elevated metabolic activity under saline or even hypersaline conditions. Such microorganisms are defined as halotolerant or halophilic species, respectively [23]. It has been documented that halophilic and halotolerant microorganisms possess the capacity to adapt to saline or hypersaline environments by employing three primary strategies to maintain osmotic pressure equilibrium within and outside the cell. These strategies encompass (1) the excessive uptake of K^+^ into microbial cells for replacing Na^+^/H^+^, (2) the intracellular synthesis and accumulation of compatible solutes, and (3) the synthesis of halophilic enzymes [24,25,26]. In order to address the issue of residual s-triazine herbicides in saline environments, a number of halotolerant/halophilic microbial strains have been isolated and investigated. For instance, a *Pseudomonas* sp. strain ADP was found to degrade more than 96% of 25 mg/L atrazine within 2 d under saline conditions (containing 30 g/L NaCl) [27]. Another bacterial strain, *Arthrobacter* sp. ZXY-2, has also been reported to degrade >50% of 100 mg/L atrazine after 30 h under saline conditions (containing 100 g/L NaCl) [28]. However, there is still a paucity of research on the biodegradation of other s-triazine herbicides (e.g., prometryne) by halotolerant or halophilic microorganisms. It is, therefore, imperative to exploit more efficient halotolerant/halophilic microbes for treating s-triazine herbicides under saline conditions.

The present study focused on the isolation and identification of halotolerant microbial strains capable of degrading s-triazine herbicides. The conditions for biodegradation of prometryne (the target compound) by growing cells of the target microbial strain were optimized through single-factor experiments. The degradation performance of some other s-triazine herbicides (atrazine, ametryne, simetryne, and cyanazine) by the target strain was also investigated and compared. Subsequently, the degradation intermediates of prometryne were identified using ultra-high-performance liquid chromatography followed by time-of-flight mass spectrometry (UHPLC-TOFMS). Meanwhile, the genome of the target strain was analyzed, and relevant functional genes involved in the biodegradation of s-triazine compounds were screened from the genome sequencing results. Based on the results of degradation intermediate detection and genome sequencing, possible degradation pathways of prometryne were proposed. Furthermore, the halotolerance mechanisms of the target strain were also discussed through screening the halotolerance-related genes from the genome. It is expected that this study will provide a potentially effective microbial strain and corresponding operation parameters for the further bioremediation of residual s-triazine herbicides in saline environments.

## 2. Materials and Methods

### 2.1. Reagents

Five s-triazine herbicides, including prometryne, atrazine, ametryne, simetryne, and cyanazine, were purchased from TCI Development Co., Ltd. (Shanghai, China), with a purity of >98.0%. The main chemical information of these s-triazine herbicides is outlined in the Supplementary Information (SI) File, Table S1. Other chemical reagents (analytical-grade) were purchased from Kemiou Chemical Reagent Co., Ltd. (Tianjin, China), and Solarbio Science & Technology Co., Ltd. (Beijing, China). Biological reagents were acquired from Sangon Biotech Co., Ltd. (Shanghai, China).

### 2.2. Experimental Design

#### 2.2.1. Isolation and Identification of the Halotolerant Prometryne-Degrading Bacterial Strain

A bacterial strain capable of efficiently degrading prometryne (the target compound) under saline conditions was isolated from the sediment of a sea cucumber seeding pond in Dalian, China. The isolation was conducted using the serial dilution and spread plate method. The liquid culture medium contained (per liter) 2.0 g KH_2_PO_4_, 0.2 g MgSO_4_, 3.3 g Na_2_HPO_4_, 30.0 g NaCl, 0.1 g yeast extract, 0.00025 g FeCl_3_, 20.00 mg prometryne, and 1.0 mL mixed solution of trace elements, which contained (per liter) 10 g ZnSO_4_, 0.5 g (NH_4_)_2_MoO_4_, 2.0 g FeSO_4_, 2.0 g MnSO_4_, and 0.8 g CuSO_4_. The initial pH was adjusted to approximately 7.0. The solid medium was prepared by adding 2% (*w*/*v*) gellan gum to the liquid medium. Both the liquid and solid media were sterilized at 121 °C for 20 min prior to use. The culturing conditions comprised a temperature of 35.0 °C and a shaking speed of 160 rpm. The target strain was identified by the 16S rDNA sequencing method.

#### 2.2.2. Prometryne Degradation by Growing Cells of the Target Strain

A fresh cell suspension of the target strain (in the exponential growth phase, with an initial OD_600_ of approximately 0.162) was inoculated into 50 mL of liquid medium containing 20.00 mg/L prometryne at an inoculum of 2% (*v*/*v*). The culture was then cultured at 35.0 °C and 160 rpm. During the cultivation process, both the concentration of residual prometryne and the density of the cell suspension were measured at 0 h, 4 h, 8 h, 9 h, 10 h, 11 h, 12 h, 13 h, 14 h, 16 h, 20 h, 24 h, 28 h, 32 h, 36 h, 40 h, 44 h, and 48 h. Meanwhile, two additional groups of media were established as controls: one uninoculated and the other inoculated with inactivated cells at the same concentration of inoculum. These controls were utilized to estimate the elimination of prometryne through auto-decomposition and bio-adsorption, respectively.

#### 2.2.3. Optimization of Conditions for Prometryne Degradation by Growing Cells of the Halotolerant Strain

The conditions for prometryne degradation by growing cells of the target strain were optimized through batch experiments performed in 150 mL shaking flasks with a working volume of 50 mL. The optimized conditions included the type of additional carbon source (yeast extract, sucrose, glucose, sodium acetate, lactose, maltose, soluble starch, methanol and ethanol, at a concentration of 0.1 g/L), concentration of the optimal additional carbon source (0–0.1 g/L), salinity (NaCl concentration, 0–30.0 g/L), initial pH (3.0–9.0), temperature (20.0–40.0 °C), rotation speed (0–200 rpm), and prometryne concentration (5.00–30.00 mg/L). In addition to the optimized parameter, the remaining parameters were set as follows: yeast extract of 0.1 g/L, NaCl of 30.0 g/L, prometryne of 20.00 mg/L, initial pH of 7.0, temperature of 35.0 °C, and rotation speed of 160 rpm, which were determined through pre-experiments. In addition, the degradation performance of five different s-triazine herbicides, including prometryne, atrazine, ametryne, simetryne, and cyanazine (at a concentration of 20.00 mg/L), was also investigated by the target strain.

#### 2.2.4. Possible Prometryne-Degrading Pathways and Halotolerance Mechanisms of the Target Strain

The degradation pathways of prometryne by the target strain were proposed based on the determination of possible degradation intermediates and the genome sequencing analysis of the strain. The degradation intermediates of prometryne were analyzed by ultra-high-performance liquid chromatography followed by time-of-flight mass spectrometry (UHPLC-TOFMS). In addition, the relevant functional genes involved in the biodegradation of s-triazine compounds were screened from the genome of the target strain. On the other hand, possible halotolerance mechanisms of the target strain were revealed by screening the halotolerance-related genes from the genome.

### 2.3. Assays

#### 2.3.1. Density of Bacterial Cell Suspension

The density of the bacterial cell suspension was determined by spectrophotometry (JASCO V-560, Tokyo, Japan) and represented by the absorbance at 600 nm (OD_600_). The supernatant of the bacterial cell suspension after centrifugation was used as the reference solution.

#### 2.3.2. Concentration of S-Triazine Herbicides

First, cell pellets and other solids were separated from the bacterial culture by centrifugation at 4.0 °C and 10,000 rpm for 10 min. The supernatant was filtered with a 0.22 μm aqueous phase filter to obtain the preliminary filtrate. Subsequently, 10.0 μL of the preliminary filtrate was transferred to a 50 mL polyethylene centrifuge tube and mixed with 10.0 mL ultrapure water, 10.0 mL acetonitrile, and 2.0 g NaCl. After shaking and extracting the mixture for 5 min, the extract was separated by centrifugation at room temperature and 9500 rpm for 5 min, and the supernatant was filtered with a 0.22 μm aqueous phase filter. The final filtrate was used for instrumental analysis.

The concentration of s-triazine herbicides was determined by high-performance liquid chromatography followed by mass spectrometry (HPLC-MS). A Waters ACQUITY (Milford, MA, USA) BEH C18 chromatographic column (2.1 mm × 50 mm, 1.7 μm) was used for the HPLC analysis at a column temperature of 30.0 °C, a flow rate of 0.3 mL/min, and a total runtime of 5 min. The mobile phase consisted of eluents A (ultrapure water containing 0.1% (*v*/*v*) formic acid) and B (acetonitrile containing 0.1% (*v*/*v*) formic acid). The gradient elution procedures were set as follows: 90% A at 0–1.0 min, 70% → 20% A at 1.0–3.0 min, and 90% A at 3.0–5.0 min. In addition, the conditions for MS analysis were as follows: the ion source was an electrospray ionization (ESI) system operating in positive ion mode with multiple reaction monitoring (MRM); ESI temperature of 150.0 °C; capillary voltage of 0.5 kV; desolvation gas (N_2_) temperature and flow rate of 500.0 °C and 800.0 L/h, respectively; cone gas flow rate of 50.0 L/h; and retention time of 0.163 s. The other parameters of the MS analysis are given in the SI File, Table S2.

#### 2.3.3. Analysis of Possible Degradation Intermediates of Prometryne

The target bacterial strain was inoculated into the liquid medium containing 20.00 mg/L prometryne and was cultivated at 35 °C and 160 rpm for 12 h. Samples were collected at 0 h, 8 h, and 12 h and pretreated by the method described in Section 2.3.2 to determine the possible prometryne degradation intermediates.

The intermediates were analyzed by ultra-high-performance liquid chromatography followed by time-of-flight mass spectrometry (UHPLC-TOFMS). A Waters ACQUITY BEH C18 chromatographic column (2.1 mm × 150 mm, 2.7 μm) was applied for the UHPLC analysis at a column temperature of room temperature and a flow rate of 0.2 mL/min. Eluents A (ultrapure water containing 0.1% (*v*/*v*) formic acid) and B (acetonitrile containing 0.1% (*v*/*v*) formic acid) served as the mobile phase in a gradient mode (95% A at 0–2.5 min, 95% → 70% A at 2.5–5 min, 70% → 20% A at 5–10 min, 20% → 95% A at 10–11 min, 95% A at 11–21 min). In addition, the TOFMS conditions were as follows: the ion source was an electrospray ionization (ESI); dry gas (N_2_) flow rate of 10 L/min; nebulizer chamber temperature and pressure of 350.0 °C and 40 psig, respectively; reference ion *m*/*z* of 112.9856 and 1033.9881; *m*/*z* scan range of 50–1100; and collision voltage of 100 V.

#### 2.3.4. Genome Sequencing

The cell suspension of the target strain was inoculated into the liquid medium containing 20.00 mg/L prometryne and cultured at 35.0 °C and 160 rpm for 12 h. The cells were then harvested by centrifugation at 4.0 °C and 10,000 rpm for 10 min, followed by three washes with 0.02 mol/L phosphate buffer (pH 7.2). Finally, the cell pellets were cryopreserved in liquid nitrogen and stored at −80.0 °C prior to genome sequencing.

Total DNA was extracted using a soil genomic DNA extraction kit (TIANGEN Biotech (Beijing) Co., Ltd., Beijing, China). DNA quality analysis, library construction, and sequencing were performed by Novogene Bioinformatics Technology Co., Ltd. (Beijing, China). Bioinformatic analysis of the sequencing results was performed according to the methods described by Layoun et al. [29]. Detailed information can be found in the SI File, Text S1.

### 2.4. Statistical Analysis

All the analytical experiments were performed in triplicate. The experimental data were analyzed by the one-way analysis of variance (ANOVA) method using Microsoft Excel 2019. A *p*-value of less than 0.05 indicates a significant difference of data between the experimental group and the control (or the other experimental group).

## 3. Results and Discussion

### 3.1. Isolation and Identification of a Halotolerant Bacterium Capable of Degrading Prometryne Efficiently

**Figure 1:**
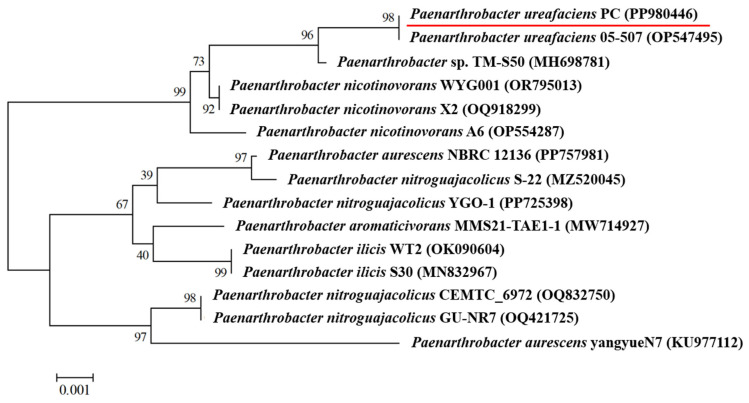
Phylogenetic tree of the strain *P. ureafaciens* PC.

A halotolerant bacterial strain capable of efficiently degrading prometryne under saline conditions (30.0 g/L NaCl) was isolated from the sediment of a sea cucumber seeding pond and was designated PC. Colonies of the strain PC that grew on the solid culture medium were milky-white, circular, slightly convex, smooth on the surface, and opaque (the SI File, Figure S1A). The strain PC was identified as a Gram-positive bacterium based on the Gram staining test (the SI File, Figure S1B). The cells of the strain PC were rod-shaped, with the mean diameter of approximately 2.8–3.4 μm × 0.43–0.49 μm (the SI File, Figure S1C). The 16S rDNA sequence of the strain PC with the length of 1465 bp was obtained and deposited in the National Center for Biotechnology (NCBI) GenBank database (https://www.ncbi.nlm.nih.gov/genbank/, accessed on 3 July 2024) with the accession number of PP980446. A phylogenetic tree of the strain PC (Figure 1) was constructed, which showed that it shared 100.0% homology with *Paenarthrobacter ureafaciens* 05-507 (OP547495). Accordingly, the strain PC was identified as *P. ureafaciens*. The strain PC was preserved in the China General Microbiological Culture Collection Center (CGMCC) under the preservation number of CGMCC 1.64779.

### 3.2. Prometryne Degradation by Growing Cells of the Strain PC

**Figure 2:**
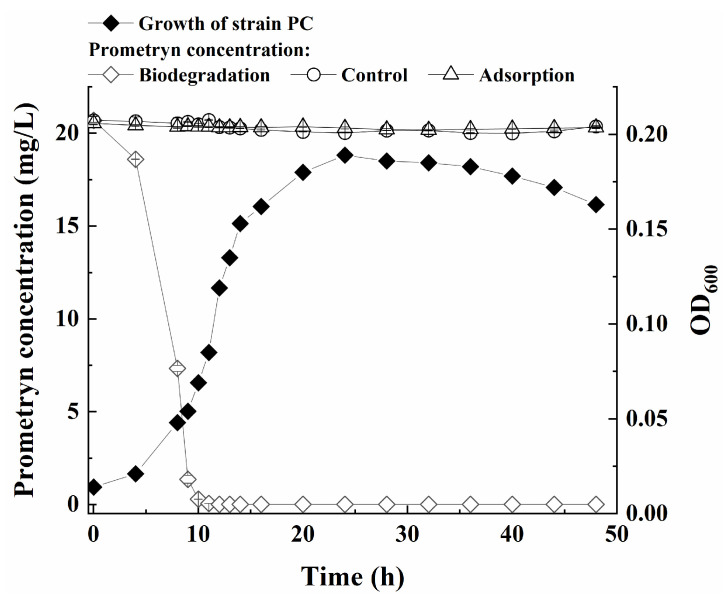
Growth of strain PC and prometryne degradation curves.

As demonstrated in Figure 2, the time curves of prometryne degradation, bacterial cell growth, and two control groups were determined. The degradation percentages of prometryne by growing cells of the strain PC at 4 h, 8 h, and 12 h were 10.1%, 64.6%, and 100.0%, respectively. Meanwhile, the strain PC entered the logarithmic phase at approximately 8 h and transitioned to the late logarithmic phase at around 14 h. In comparison, no significant decline in prometryne concentration was observed in either the uninoculated medium or that inoculated with inactivated bacterial cells during the entire 48 h period. It was hypothesized that the elimination of prometryne was predominantly attributable to biodegradation by the strain PC.

### 3.3. Optimization of Conditions for Prometryne Degradation by Growing Cells of the Strain PC

**Figure 3:**
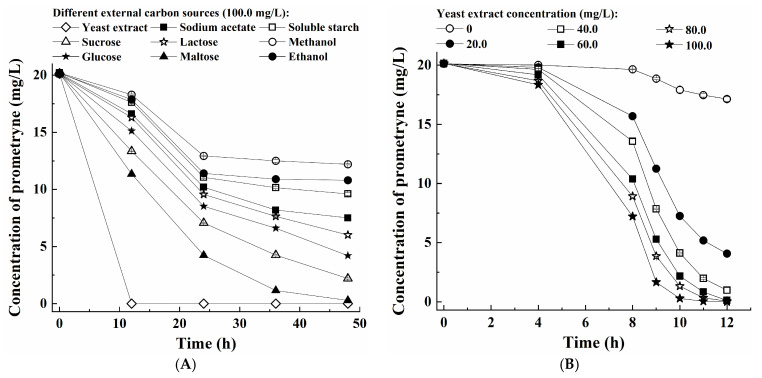

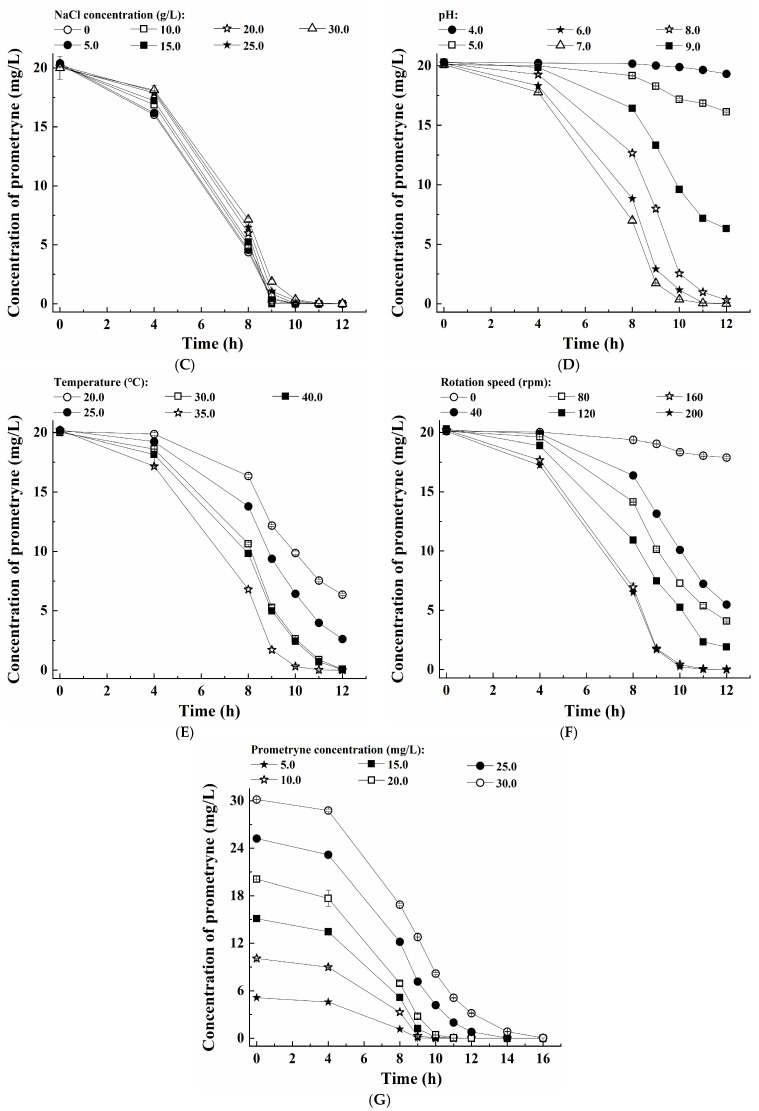
Optimization of the conditions for prometryne degradation by growing cells of the strain PC: (**A**) type of additional carbon source, (**B**) yeast extract concentration, (**C**) NaCl concentration, (**D**) pH, (**E**) temperature, (**F**) rotation speed, and (**G**) initial prometryne concentration.

As shown in Figure 3A and Figure S2A, the strain PC exhibited a significantly diminished prometryne degradation efficiency and cell growth rate when using ethanol or methanol as the additional carbon source. Less than 50.0% of 20.00 mg/L prometryne was degraded within 48 h. In comparison, the addition of sucrose, glucose, lactose, soluble starch, sodium acetate, or maltose resulted in higher prometryne degradation percentages within 48 h (ranging from 52.4% to 98.6%) as well as faster cell growth rates of the strain PC. Furthermore, yeast extract was found to have the greatest promotive effect on both prometryne degradation and the growth of the strain PC. Growing cells of the strain PC were capable of completely degrading 20.00 mg/L prometryne within 12 h using 0.1 g/L yeast extract as the co-substrate. Consequently, yeast extract was selected as the optimal additional carbon source for further study. In addition, the strain was found to be capable of using single carbon sources (e.g., maltose, sucrose, glucose, etc.) for both prometryne degradation and cell growth, suggesting that the strain PC was able to utilize prometryne as the sole nitrogen source because prometryne was the only nitrogenous component in the medium. Subsequently, the effect of the yeast extract concentration on the strain PC was further investigated. As demonstrated in Figure 3B, the degradation efficiency of prometryne increased significantly with the presence of 0.02–0.1 g/L yeast extract in comparison to the absence of yeast extract. Meanwhile, the cell growth rate of the strain PC was also significantly enhanced by the incorporation of 0.02–0.1 g/L yeast extract (Figure S2B). To ensure the complete degradation of 20.00 mg/L prometryn within 12 h, the optimal concentration of additional yeast extract was determined to be 0.1 g/L.

Figure 3C and Figure S2C show the impact of salinity (NaCl concentration) on the prometryne degradation efficiency and growth rate of the strain PC. When the NaCl concentration ranged from 0 g/L to 10.0 g/L, the strain PC was capable of completely degrading 20.00 mg/L prometryne within 10 h. As the NaCl concentration increased to 15.0–20.0 g/L, 100% prometryne degradation percentages were also achieved when the culturing time was extended to 11 h. When the NaCl concentration was further increased to 25.0–30.0 g/L, the least time for complete degradation of 20.00 mg/L prometryne was 12 h. It was suggested that the strain PC could maintain relatively high metabolic activity under saline conditions. However, it was observed that the solubility of prometryne decreased significantly when the NaCl concentration was increased beyond 30.0 g/L. Consequently, the impact of NaCl concentrations exceeding 30.0 g/L on the strain PC was not addressed in this study. Meanwhile, higher salinity appeared to inhibit the growth of the strain PC. These findings suggest that the strain PC possessed a certain degree of tolerance to saline conditions, but it did not depend on high salinity for its survival. Consequently, it could be concluded that the strain PC was a halotolerant bacterium rather than a halophilic one [27].

As shown in Figure 3D–F, approximately 20.00 mg/L prometryne was completely degraded within 12 h under the following conditions: pH of 7.0, temperature of 35.0 °C, and rotation speed of ≥160 rpm. Meanwhile, the strain PC also exhibited the fastest growth rate under the same conditions (Figure S2D–F). Therefore, the optimal pH, temperature, and rotation speed were determined to be 7.0, 35.0 °C, and 160 rpm, respectively. It was deduced that the strain PC was a typical aerobic and mesophilic bacterium that exhibited a preference for neutral pH conditions.

Finally, the effect of the initial prometryne concentration on the prometryne-degradation performance and cell growth of the strain PC was also investigated (Figure 3G and Figure S2G). The results demonstrated that a range of 5.00–20.00 mg/L prometryne could be completely degraded within 10–12 h. When the initial prometryne concentration increased to 25.00 mg/L, the degradation percentage was also higher than 96.0% within 12 h and achieved 100.0% within 16 h. Even when the initial prometryne concentration was further increased to approximately 30.00 mg/L, the corresponding degradation percentage could also be higher than 99.0% within 16 h. To provide a comprehensive analysis of the degradation rate of prometryne by the strain PC, the average specific degradation rate was calculated and represented as the amount of prometryne degraded by unit bacterial cell per unit time. As demonstrated in Figure S3, an increase in the initial prometryne concentration resulted in a concomitant rise in the average specific degradation rate. This phenomenon can be attributed to either an escalation in secretion levels or heightened activity of key enzymes involved in prometryne biodegradation [30]. Meanwhile, the growth rate of the strain PC was found to be reduced as a consequence of an elevated toxicity level, attributable to elevated levels of prometryne.

**Figure 4:**
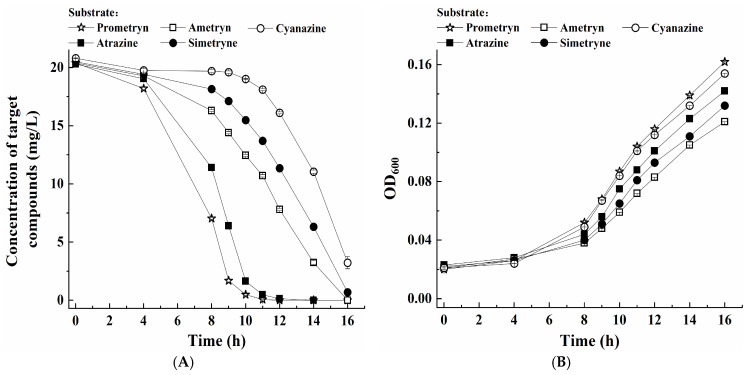
(**A**) Degradation of five different s-triazine herbicides by growing cells of the strain PC and (**B**) corresponding bacterial growth.

Furthermore, this study investigated the degradation of four additional s-triazine herbicides, along with prometryne, by growing cells of the strain PC under optimal conditions. The results demonstrated that growing cells of the strain PC efficiently degraded five s-triazine herbicides (20.00 mg/L), with the maximal degradation percentages ranging from 84.5% to 100.0% within 12–16 h (Figure 4A). Among these, prometryne was the most rapidly degraded (100.0% within 12 h), followed by atrazine, ametryne, simetryne, and cyanazine. On the other hand, the five s-triazine herbicides exhibited different effects on the cell growth of the strain PC (Figure 4B). The fastest growth rate of the strain PC was also achieved during the biodegradation of prometryne.

### 3.4. Speculation of Degradation Pathways of Prometryne by the Strain PC

#### 3.4.1. Possible Degradation Intermediates of Prometryne by the Strain PC

As discussed above, the degradation of prometryne by the strain PC was predominantly attributed to biodegradation. In order to speculate on possible degradation pathways of prometryne by the strain PC, degradation intermediates were identified by the UHPLC-TOFMS method. The results (in the SI File, Figure S4) showed that after degradation for 8 h, four possible intermediates were identified as 4,6-bis(isopropylamino)-1,3,5-triazin-2-ol, 6-isopropylamino-1,3,5-triazine-2,4-diol, N2-isopropyl-6-methylthio-1,3,5-triazine-2,4-diamine, and 4-amino-6-isopropylamino-1,3,5-triazine-2-ol, corresponding to the *m*/*z* (ion peaks) of 212.1511, 171.0876, 200.0962, and 170.1040, respectively. Following a 12 h degradation period, only N2-isopropyl-6-methylthio-1,3,5-triazine-2,4-diamine and 4-amino-6-isopropylamino-1,3,5-triazine-2-ol were identified (see the SI File, Figure S5). It was hypothesized that 4,6-bis(isopropylamino)-1,3,5-triazin-2-ol and 6-isopropylamino-1,3,5-triazin-2,4-diol were entirely transformed into secondary products during the 8th to the 12th hour.

#### 3.4.2. Genome Sequencing of the Strain PC

In addition to the detection of degradation intermediates, the genome of the strain PC was subjected to sequencing and analysis in order to acquire further information for proposing possible degradation pathways of prometryne. The results showed that after the quality control of the raw DNA sequences of the strain PC, 10,773,192 clean reads were obtained, with an average GC content of 62.87%. The average Q20 and Q30 percentages were 97.77% and 93.69%, respectively. A total of 4297 coding genes were predicted, among which 2111 and 3209 genes were successfully annotated in the KEGG and the COG databases, respectively (see the SI File, Figure S6).

Based on the available literature on functional genes involved in the biodegradation of s-triazine herbicides, genes that might be related to prometryne degradation were screened from the genome of the strain PC. The results (Table 1) showed that a gene *hapE*, which was annotated in the KEGG database, was identified. Liang et al. [4] reported that prometryne was transformed into 4,6-bis(isopropylamino)-1,3,5-triazin-2-ol by *Pseudomonas* sp. DY-1 through the catalysis of 4-hydroxyacetophenone monooxygenase, which was encoded by *hapE*. Consequently, it was proposed that *hapE* might also be responsible for the initial step of prometryne degradation by the strain PC. Meanwhile, another gene encoding laccase, which was annotated in the COG database, was also identified (Table 2). It has been reported that laccase typically displays elevated catalytic activity and a broad range of substrate diversity [31]. For instance, the hydrolytic dechlorination of atrazine was shown to be catalyzed by laccase [31]. Meanwhile, the transformation of 4-ethylamino-6-isopropylamino-1,3,5-triazine-2-ol into cyanuric acid was also catalyzed by laccase [31]. It was hypothesized that the demethylthiolation and deisopropylamination of prometryne might also be catalyzed by laccase due to the similarity in chemical structure between prometryne and atrazine. Furthermore, a gene (*guaD*) encoding guanine deaminase was identified. As previously reported, ammeline was transformed into ammelide through hydrolytic deamination, which was catalyzed by guanine deaminase [32]. It was hypothesized that, given the similarity in chemical structure between 4-amino-6-isopropylamino-1,3,5-triazine-2-ol and ammeline, the deamination of 4-amino-6-isopropylamino-1,3,5-triazine-2-ol might also be catalyzed by guanine deaminase (encoded by *guaD*). Another study showed that cyanuric acid was transformed into 1-carboxybiuret through the opening of the triazine ring, which was catalyzed by cyanuric acid amidohydrolase encoded by the gene *atzD* [17]. However, *atzD* was not annotated in the genome of the strain PC, which might be due to incomplete information in the relevant databases or the possibility that the process was catalyzed by other unconfirmed and unreported genes. Furthermore, two genes, *gatA* and *gatC*, which encode glutamine-dependent amidotransferase subunits A (GatA) and C (GatC), respectively, were identified in the genome. It was reported that the catalytic substrate of the 1-carboxybiuret hydrolase (AtzE) and a small protein that is required for soluble expression of AtzE (AtzG) share a similar structure with that of GatA and GatC, respectively [17,33]. It was hypothesized that GatA and GatC might share the same catalytic function with AtzE and AtzG, respectively. According to Esquirol et al. [17], 1-carboxybiuret was transformed into urea-1,3-dicarboxylate with the catalysis of AtzE and AtzG. Therefore, it was proposed that the intermediate 1-carboxybiuret might be transformed into urea-1,3-dicarboxylate through the catalysis of GatA and GatC (encoded by *gatA* and *gatC*, respectively) during prometryne degradation by the strain PC. In addition, a gene (*biuH*) encoding biuret amidohydrolase, which was annotated in the KEGG database, and six additional genes (encoding allophanate hydrolase subunits 1 and 2), which were annotated in the COG database, were screened out. According to Cameron et al. [34], biuret was transformed into allophanate with the catalysis of biuret amidohydrolase. Subsequent to this, allophanate was finally mineralized to ammonia and carbon dioxide through the catalysis of allophanate hydrolase, which was encoded by the gene *atzF* [35]. Therefore, it was hypothesized that the seven genes encoding biuret amidohydrolase and allophanate hydrolase might be responsible for the final two steps of prometryne mineralization.

#### 3.4.3. Possible Degradation Pathways of Prometryne by the Strain PC

**Figure 5:**
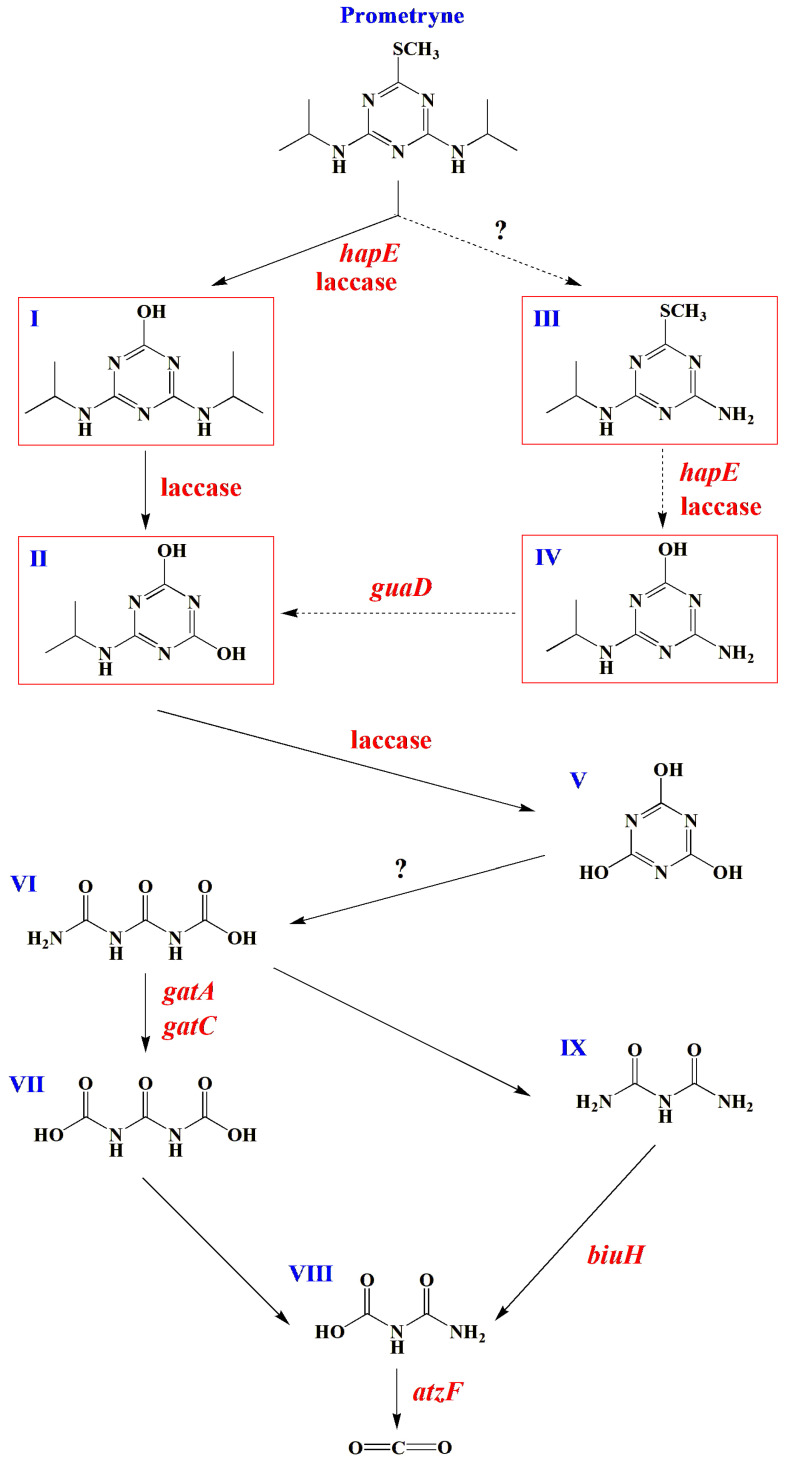
Possible prometryne-degrading pathways by the strain PC: (**I**), 4,6-bis(isopropylamino)-1,3,5-triazin-2-ol; (**II**), 6-isopropylamino-1,3,5-triazine-2,4-diol; (**III**), N2-isopropyl-6-methylthio-1,3,5-triazine-2,4-diamine; (**IV**), 4-amino-6-isopropylamino-1,3,5-triazin-2-ol; (**V**), cyanuric acid; (**VI**), 1-carboxybiuret; (**VII**), urea-1,3-dicarboxylate; (**VIII**), allophanate; (**IX**), biuret. The prometryne degradation intermediates detected by UHPLC-TOFMS are indicated by red boxes.

Following a comprehensive analysis of prometryne-degradation intermediates and genome sequencing, a number of possible degradation pathways for prometryne by the strain PC were proposed (Figure 5). As reported, the initial step in prometryne biodegradation is typically the hydrolytic demethylthiolation process [13]. It was thus proposed that prometryne undergoes a series of metabolic transformations, beginning with the formation of 4,6-bis(isopropylamino)-1,3,5-triazin-2-ol (compound I). This process might be catalyzed by laccase and/or 4-hydroxyacetophenone monooxygenase (encoded by *hapE*). Subsequently, compound I might be transformed into 6-isopropylamino-1,3,5-triazine-2,4-diol (compound II) through hydrolytic deisopropylamination, which might also be catalyzed by laccase. In addition, prometryne could also be initially transformed into N2-isopropyl-6-methylthio-1,3,5-triazine-2,4-diamine (compound III) through hydrolytic deisopropylamination [36]. However, no gene encoding the definitive enzymes related to this process was identified during the screening process. Then, compound III might be transformed into 4-amino-6-isopropylamino-1,3,5-triazin-2-ol (compound IV) through hydrolytic deisopropylamination, which also might be catalyzed by laccase and/or 4-hydroxyacetophenone monooxygenase (encoded by *hapE*). Furthermore, compound IV might be transformed into compound II through hydrolytic deamination, which might be catalyzed by guanine deaminase (encoded by *guaD*). The above steps (from compounds III through IV to II) were the same as those reported by Esquirol et al. [35]. The preceding analysis indicates that compound II was generated in both of the two potential prometyne-degrading upstream pathways. Consequently, it is plausible that compound II underwent transformation into cyanuric acid (compound V) through the hydrolytic deisopropylamination process, a reaction that might be catalyzed by laccase [31]. It was hypothesized that compound V, which was also the intermediate during the biodegradation of atrazine, might be transformed into 1-carboxybiuret (compound VI) through the opening of the triazine ring [35]. However, this step remains to be further confirmed since the corresponding gene *atzD* was not annotated in any of the commonly used databases. The subsequent transformation of compound VI into urea-1,3-dicarboxylate (compound VII) was identified as a possibility, catalyzed by GatA and GatC (encoded by *gatA* and *gatC*, respectively). Esquirol et al. [35] hypothesized that compound VII might undergo spontaneous hydrolytic decarboxylation to form allophanate (compound VIII). Furthermore, it was demonstrated that compound VI might undergo spontaneous decarboxylation, resulting in the formation of biuret (compound IX). Subsequent to this, compound IX might be subject to hydrolytic deamination, a process that might be catalyzed by biuret amidohydrolase (encoded by *biuH*). The degradation pathway from compounds VI through IX to VIII was found to be analogous to that reported by Esquirol et al. [37]. Furthermore, it was hypothesized that compound VIII might undergo mineralization to ammonia and carbon dioxide, a process that might be catalyzed by allophanate hydrolase (encoded by *atzF*). In this study, four possible intermediates (compounds I, II, III, and IV) generated in the upstream degradation pathway of prometryne were detected. However, the intermediates in the downstream pathway, including compounds V, VI, VII, VIII, and IX, could not be identified. This might be attributed to the hypothesis that these downstream metabolites, which possess a lower molecular weight compared to their upstream counterparts, might undergo rapid mineralization.

### 3.5. Possible Halotolerance Mechanisms of the Strain PC

Halotolerant and halophilic microorganisms are capable of surviving and maintaining normal physiological and metabolic activities in saline environments by means of specific adaptation mechanisms [38]. In order to discuss and reveal possible halotolerance mechanisms of the strain PC, halotolerance-related genes were screened from the genome sequencing results based on the relevant literature.

As reported, halotolerant and halophilic microorganisms could adapt to saline conditions through the simultaneous uptake of excessive K^+^ into cells and excretion of Na^+^/H^+^ by ion transporters, which is generally defined as the “hypersaline-in” strategy [26]. The currently identified K^+^ transporters predominantly belong to the Trk, Ktr, Kdp, and Kup families [39]. A comprehensive screening of the genome of the strain PC identified numerous genes encoding K+ transporters, including those encoding *trkA*, *trkG*, *trkH*, *ktrA*, *ktrB*, *ktrC*, and *ktrD*, which were responsible for K^+^ uptake by the Trk/Ktr system [40]. As reported by Guo et al. [41], the protein TrkA can interact with TrkH to promote the transport of K^+^. Furthermore, KtrA has been observed to bind to the transmembrane protein KtrB, thereby forming a complex that facilitates the transportation of K^+^ [42]. Meanwhile, KtrA and KtrB exhibited high levels of similarity with KtrC and KtrD, respectively [43]. On the other hand, numerous Na^+^/H^+^ antiporters were also responsible for the halotolerance of microorganisms, including the members of the cation/proton antiporter 1, 2, and 3 (CPA-1, CPA-2, and CPA-3) families [44]. In this study, several genes related to Na^+^/H^+^ antiporters were identified in the genome of the strain PC, including *nhaK*, belonging to the CPA-1 family; *nhaA*, belonging to the CPA-2 family; and *mrpA*, *mrpC*, *mrpD*, *mrpE*, *mrpF*, and *mrpG*, belonging to the CPA-3 family. It was reported that NhaK, a sodium efflux transporter belonging to the CPA-1 family, played a significant role in resisting high salinity for two halotolerant bacteria: *Staphylococcus aureus* and *Bacillus subtilis* [45,46]. Furthermore, some ion transporters belonging to the CPA-2 family have been confirmed to be involved in adapting to saline environments [47]. For instance, NhaA, belonging to the CPA-2 family, was the first Na^+^/H^+^ antiporter isolated from bacteria [48]. An additional ion antiporter, NhaA, was shown to be instrumental in maintaining the osmotic pressure equilibrium between microbial cells and their external environment under saline conditions, as evidenced by the experimental transfer of the encoding gene (*nhaA*) into *Escherichia coli* KNabc [49]. In addition, another ion transporter, Mrp, belonging to the CPA-3 family, was first identified in a halotolerant *Bacillus halotolerans* strain [50]. Mrp proteins generally exist in the form of complexes, with MrpA and MrpD as two of the primary subunits [51,52]. Furthermore, MrpA and MrpD were found to be responsible for the antiport of Na^+^ and H^+^, respectively [51]. The above analysis suggested that the “hypersaline-in” strategy might be one of the significant halotolerance mechanisms of the strain PC.

On the other hand, halotolerant and halophilic microorganisms could also tolerate high-osmotic environments through the production and intracellular accumulation of compatible solutes such as polyols, amino acids, sugars, and methylamines, which was generally defined as the “organic-solutes-in” strategy [53]. As reported, glycerol was a typical compatible solute [54]. Meanwhile, halotolerant/halophilic microorganisms could convert glucose into glycerol by the catalysis of glycerol-3-phosphate dehydrogenase [55]. In this study, several genes encoding glycerol-3-phosphate dehydrogenase, including *gpsA*, *glpA*, and *glpD*, were identified through the screening of the genome. It was proposed that glycerol might be one of the compatible solutes of the strain PC. In addition, glutamate and glutamine, which were found to be accumulated in the cells of many halotolerant and moderately halophilic microorganisms, could be synthesized through the coordinated catalysis of glutamate synthase, glutamate dehydrogenase, and glutamine synthetase [56]. Five genes encoding glutamate synthase (*gltB* and *gltD*), glutamate dehydrogenase (*gdhA*), and glutamine synthetase (*glnA* and *glnE*) were screened out, suggesting that glutamate and glutamine might also be the compatible solutes of the strain PC. Trehalose was also a compatible solute that could be synthesized by trehalose-6-phosphate synthase/phosphatase, according to Cardoso et al. [57]. Two genes, *otsA* and *otsB*, encoding trehalose-6-phosphate synthase and trehalose-6-phosphate phosphatase, respectively, were also screened out from the genome. It was suggested that the strain PC could also tolerate high salinity through intracellular synthesis and accumulation of trehalose. Similarly, betaine, another available compatible solute for halotolerant/halophilic microorganisms [58], could be synthesized from choline through the catalysis of glycine betaine monooxygenase [59]. Accordingly, two genes encoding glycine betaine monooxygenase (*bmoA* and *bmoB*) were screened out, suggesting that betaine might be another compatible solute for the strain PC. On the other hand, the accumulation of compatible solutes in cells of halotolerant/halophilic microorganisms could also be mediated by the corresponding transporters. Accordingly, the genes encoding trehalose/maltose transporters (*thuE*, *thuF*, and *thuG*) [60] and proline/betaine transporters (*proP* and *betS*) [58,61] were determined. The above results suggested that the strain PC might also tolerate saline conditions through the intracellular accumulation of various possible compatible solutes.

The production of halophilic enzymes has been reported as another possible halotolerance mechanism for halotolerant/halophilic microorganisms [25]. Two genes encoding ribonucleases (*rnhA* and *rnhB*) belonging to halophilic enzymes were identified in the genome [62]. As reported, the surface of halophilic enzymes was enriched in negative charges provided by acidic amino acids [25], which contributed to the increase in protein solubility under saline conditions [63]. Therefore, the production of halophilic enzymes could also be one of the important halotolerance mechanisms of the strain PC.

**Figure 6:**
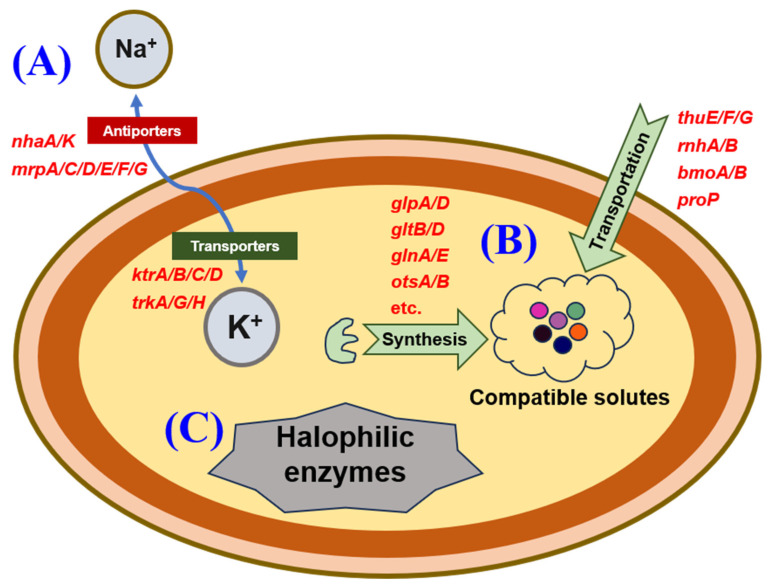
A schematic diagram of the possible halotolerance mechanisms of strain PC: (**A**) the “hypersaline-in” strategy, (**B**) the “organic-solutes-in” strategy, and (**C**) the production of halophilic enzymes.

In summary, the possible halotolerance mechanisms of the strain PC mainly included (1) the simultaneous excessive uptake of K^+^ into cells and excretion of redundant Na^+^ and H^+^ out of cells (the “hypersaline-in” strategy); (2) the intracellular synthesis or uptake of organic compatible solutes (the “organic-solutes-in” strategy); and (3) the production of halophilic enzymes (as shown in Figure 6).

## 4. Conclusions

A halotolerant strain capable of degrading prometryne and tolerating 30.0 g/L NaCl was isolated and identified as *P. ureafaciens* PC. Growing cells of the strain PC could completely degrade 20.00 mg/L prometryne within 12 h under the following conditions: addition of 0.1 g/L yeast extract as the additional carbon source, initial pH of 7.0, temperature of 35 °C, and rotation speed of ≥160 rpm. Four possible degradation intermediates of prometryne were identified as 4,6-bis(isopropylamino)-1,3,5-triazin-2-ol, 6-isopropylamino-1,3,5-triazine-2,4-diol, N2-isopropyl-6-methylthio-1,3,5-triazine-2,4-diamine, and 4-amino-6-isopropylamino-1,3,5-triazine-2-ol. Furthermore, a comprehensive screening of the genome of the strain PC identified related functional genes, including *hapE*, *guaD*, *gatA*, *gatC*, *biuH*, and *atzF*, and another one encoding laccase. The preliminary findings, based on the identification of potential degradation intermediates and comprehensive genome analysis, suggested that prometryne might be degraded by the strain PC through a series of steps, including hydrolytic demethylthiolation, hydrolytic deisopropylamination, hydrolytic deamination, triazine ring opening, hydrolytic decarboxylation, etc. Furthermore, the genes encoding Na^+^/H^+^ antiporters, K^+^ transporters, synthetases and transporters of compatible solutes, and halophilic enzymes were also screened out from the genome, which suggested that the strain PC could tolerate high salinity through the “hypersaline-in” and “organic-solutes-in” strategies, as well as by producing halophilic enzymes.

## Figures and Tables

**Table 1:** Prometryn degradation-related genes of the strain PC annotated using the KEGG and COG databases.

Gene ID	Database	Functional Annotation	Gene Name	Ko/COG ID	E-Value
GM002825	KEGG	4-Hydroxyacetophenone monooxygenase	*hapE*	K14520	0.00
GM000956	KEGG	Guanine deaminase	*guaD*	K01487	4.50 × 10^−88^
GM002083	KEGG	Guanine deaminase	*guaD*	K01487	7.90 × 10^−88^
GM001055	KEGG	Aspartyl-tRNA (Asn)/glutamyl-tRNA (Gln) amidotransferase subunit A	*gatA*	K02433	1.10 × 10^−289^
GM001056	KEGG	Aspartyl-tRNA (Asn)/glutamyl-tRNA (Gln) amidotransferase subunit C	*gatC*	K02435	4.00 × 10^−47^
GM002811	KEGG	Biuret amidohydrolase	*biuH*	K23359	2.70 × 10^−134^
GM002397	COG	Copper oxidase (laccase) domain	None	COG1496	6.90 × 10^−78^
GM001342	COG	Allophanate hydrolase subunit 1	None	COG2049	8.80 × 10^−142^
GM002988	COG	Allophanate hydrolase subunit 1	None	COG2049	1.20 × 10^−256^
GM003301	COG	Allophanate hydrolase subunit 1	None	COG2049	2.30 × 10^−108^
GM001343	COG	Allophanate hydrolase subunit 2	None	COG1984	3.20 × 10^−137^
GM001611	COG	Allophanate hydrolase subunit 2	None	COG1984	1.30 × 10^−88^
GM003302	COG	Allophanate hydrolase subunit 2	None	COG1984	7.00 × 10^−172^

**Table 2:** Halotolerance-related genes of the strain PC annotated using the KEGG database.

Description	Gene ID	Functional Annotation	Gene Name	Ko ID	E-Value
The “hypersaline-in” strategy	GM000608	Monovalent cation/hydrogen antiporter	*nhaK*	K24163	4.20 × 10^−260^
	GM001133	Na^+^: H^+^ antiporter, NhaA family	*nhaA*	K03313	5.40 × 10^−229^
	GM001783	Na^+^: H^+^ antiporter, NhaA family	*nhaA*	K03313	4.40 × 10^−234^
	GM002340	Multicomponent Na^+^: H^+^ antiporter subunit A	*mrpA*	K05565	0.00
	GM002339	Multicomponent Na^+^: H^+^ antiporter subunit C	*mrpC*	K05567	3.30 × 10^−81^
	GM002338	Multicomponent Na^+^: H^+^ antiporter subunit D	*mrpD*	K05568	2.40 × 10^−279^
	GM002337	Multicomponent Na^+^: H^+^ antiporter subunit E	*mrpE*	K05569	3.40 × 10^−95^
	GM002336	Multicomponent Na^+^: H^+^ antiporter subunit F	*mrpF*	K05570	3.30 × 10^−35^
	GM002335	Multicomponent Na^+^: H^+^ antiporter subunit G	*mrpG*	K05571	4.90 × 10^−61^
	GM000828	Trk/Ktr system potassium uptake protein	*trkA*, *ktrA*, *ktrC*	K03499	4.50 × 10^−112^
	GM002457	Trk/Ktr system potassium uptake protein	*trkA*, *ktrA*, *ktrC*	K03499	6.00 × 10^−132^
	GM002458	Trk/Ktr system potassium uptake protein	*trkA*, *ktrA*, *ktrC*	K03499	3.30 × 10^−121^
	GM000827	Trk/Ktr system potassium uptake protein	*trkG*, *trkH*, *ktrB*, *ktrD*	K03498	4.30 × 10^−259^
	GM001785	Trk/Ktr system potassium uptake protein	*trkG*, *trkH*, *ktrB*, *ktrD*	K03498	2.00 × 10^−203^
	GM003781	Trk/Ktr system potassium uptake protein	*trkG*, *trkH*, *ktrB*, *ktrD*	K03498	2.40 × 10^−230^
The “organic-solutes-in” strategy	GM000083	Glycerol-3-phosphate dehydrogenase (NAD(P)+)	*gpsA*	K00057	1.00 × 10^−185^
	GM000457	Glycerol-3-phosphate dehydrogenase	*glpA*, *glpD*	K00111	0.00
	GM002023	Glycerol-3-phosphate dehydrogenase	*glpA*, *glpD*	K00111	0.00
	GM002508	Glutamate synthase (NADPH) large chain	*gltB*	K00265	0.00
	GM002509	Glutamate synthase (NADPH) small chain	*gltD*	K00266	6.60 × 10^−284^
	GM000823	Glutamate dehydrogenase (NADP+)	*gdhA*	K00262	9.00 × 10^−252^
	GM001516	Glutamine synthetase	*glnA*	K01915	4.30 × 10^−250^
	GM002413	Glutamine synthetase	*glnA*	K01915	3.30 × 10^−261^
	GM002418	Glutamine synthetase	*glnA*	K01915	1.70 × 10^−279^
	GM003074	Glutamine synthetase	*glnA*	K01915	7.60 × 10^−272^
	GM002414	Glutamine synthetase adenylyltransferase	*glnE*	K00982	0.00
	GM003815	Trehalose 6-phosphate synthase	*otsA*	K00697	8.90 × 10^−292^
	GM003814	Trehalose 6-phosphate phosphatase	*otsB*	K01087	1.00 × 10^−140^
	GM001556	Trehalose/maltose transport system substrate-binding protein	*thuE*	K10236	3.30 × 10^−234^
	GM002154	Trehalose/maltose transport system substrate-binding protein	*thuE*	K10236	5.50 × 10^−237^
	GM001555	Trehalose/maltose transport system permease protein	*thuF*	K10237	2.50 × 10^−184^
	GM002153	Trehalose/maltose transport system permease protein	*thuF*	K10237	4.80 × 10^−149^
	GM001554	Trehalose/maltose transport system permease protein	*thuG*	K10238	3.70 × 10^−160^
	GM002152	Trehalose/maltose transport system permease protein	*thuG*	K10238	1.80 × 10^−154^
	GM001511	Glycine betaine monooxygenase A	*bmoA*	K00479	5.40 × 10^−261^
	GM001510	Glycine betaine monooxygenase B	*bmoB*	K21832	3.80 × 10^−268^
	GM001698	MFS transporter, MHS family, proline/betaine transporter	*proP*	K03762	1.20 × 10^−305^
	GM002272	MFS transporter, MHS family, proline/betaine transporter	*proP*	K03762	1.40 × 10^−240^
	GM003303	MFS transporter, MHS family, proline/betaine transporter	*proP*	K03762	1.40 × 10^−240^
	GM003596	MFS transporter, MHS family, proline/betaine transporter	*proP*	K03762	8.20 × 10^−244^
Halophilic enzymes	GM001455	Ribonuclease HI	*rnhA*	K03469	5.40 × 10^−171^
	GM000017	Ribonuclease HII	*rnhB*	K03470	2.10 × 10^−140^

## Data Availability

All data generated or analyzed during this study are included in this published article and its Supplementary Information (SI) Files. The 16S rDNA sequence of *P. ureafaciens* PC was deposited to the GenBank database with the accession number of PP980446. The genome sequencing data (frame diagram) of *P. ureafaciens* PC were uploaded to the Genome Database (https://submit.ncbi.nlm.nih.gov/subs/genome/, accessed on 16 July 2024) of the National Center for Biotechnology Information (NCBI) under the accession number of JBFNQT000000000.

## Supplementary Materials

The following supporting information can be downloaded at https://www.mdpi.com/article/10.3390/microorganisms13030649/s1. Table S1: Main chemical information of five s-triazine herbicides used in this study; Table S2: Parameters for MS (quantitative) analysis of five s-triazine herbicides; Figure S1: (A) Colonial morphology, (B) Gram staining result and (C) scanning electron microscopic (SEM) micrograph of the strain PC; Figure S2: Optimization of the conditions for cell growth of the strain PC: (A) type of additional carbon source, (B) yeast extract concentration, (C) NaCl concentration, (D) pH, (E) temperature, (F) rotation speed, and (G) initial prometryne concentration; Figure S3: The average specific degradation rates of prometryne at different initial concentrations; Figure S4: TOFMS spectrums of four possible intermediates of prometryne after degradation by the strain PC for 8 h: (A) 4,6-diisopropylamino-1,3,5-triazine-2-ol; (B) 6-isopropylamino-1,3,5-triazine-2,4-diol; (C) N2- isopropyl-6-methylthio-1,3,5-triazine-2,4-diamine; (D) 4-amino-6-isopropylamino-1,3,5-triazine-2-ol; Figure S5: TOFMS spectrums of two possible intermediates of prometryne after degradation by the strain PC for 12 h: (A) N2-isopropyl-6-methylthio-1,3,5-triazine-2,4-diamine; (B) 4-amino-6-isopropylamino-1,3,5-triazine-2-ol; Figure S6: The results of gene function annotation of the strain PC based on (A) COG and (B) KEGG databases; Text S1: Genome sequencing method. References [64,65,66,67,68,69,70,71,72,73,74,75,76,77,78,79,80,81,82,83,84,85,86] are included in the Supplementary Materials.
