# Supplementary material for: Biodegradation of S-Triazine Herbicides Under Saline Conditions by Paenarthrobacter ureafaciens PC, a New Halotolerant Bacterial Isolate: Insights into Both the Degradative Pathway and Mechanisms of Tolerance to High Salt Concentrations
Source: Microorganisms. 2025 Mar 12;13(3):649. doi: 10.3390/microorganisms13030649 (PMC11945447; doi:10.3390/microorganisms13030649)
Supplement: Supplementary file 1 [file microorganisms-13-00649-s001.zip › microorganisms-3487327-supplementary.pdf]

Supporting Information for:

Biodegradation of S-Triazine Herbicides Under Saline Conditions by *Paenarthrobacter ureafaciens* PC, a New Halotolerant Bacterial Isolate: Insights into Both the Degradative Pathway and Mechanisms of Tolerance to High Salt Concentrations

Chunqing Fu [1], Yifan Jiang [1], Bingwen Xu [2], Xinmei Fu [3], Liang Tan [1,*] and Mei Jin [1,*]

[1] School of Life Science, Liaoning Normal University, Dalian 116081, China
[2] Dalian Center for Certification and Food and Drug Control, Technology Innovation Center of Rapid Screening and Traceability for Edible Agricultural Product Safety, State Administration for Market Regulation, Dalian 116037, China
[3] School of Chemical Engineering, Dalian University of Technology, Dalian 116024, China
* Correspondence: tanliang1210@lnnu.edu.cn (L.T.); jm6688210@163.com (M.J.); Tel./Fax: +86-411-85827068 (L.T. & M.J.)

This 17-page file includes:

2 Supplemental Tables

6 Supplemental Figures

1 Supplemental Texts

23 References

Table S1 Main chemical information of five s-triazine herbicides used in this study.

Name	CAS number	Chemical structure	Molecular formula
Prometryne	7287-19-6	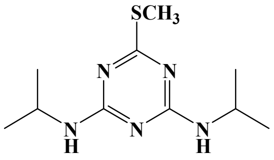	$C_{10}H_{19}N_5S$
Atrazine	1912-24-9	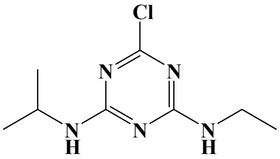	$C_8H_{14}ClN_5$
Ametryne	834-12-8	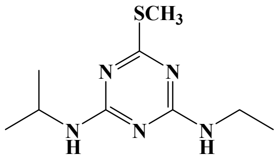	$C_9H_{17}N_5S$
Simetryne	1014-70-6	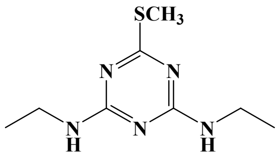	$C_8H_{15}N_5S$
Cyanazine	21725-46-2	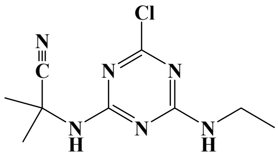	$C_9H_{13}ClN_6$

Table S2 Parameters for MS (quantitative) analysis of five s-triazine herbicides.

Compound	Solvent	Cone voltage (V)	Object ion (m/z)		
			Parent ion	Daughter ion (Corresponding collision energy, eV)	
				Quantification	Characterization
Prometryne	Acetone	40.0	242.2	158.0 (25.0)	200.1 (15.0)
Atrazine		39.0	216.1	174.1 (18.0)	96.1 (22.0)
Ametryne		38.0	228.1	186.1 (18.0)	68.1 (36.0)
Simetryne		50.0	214.0	124.0 (20.0)	95.9 (25.0)
Cyanazine		41.0	241.1	214.0 (18.0)	96.0 (30.0)

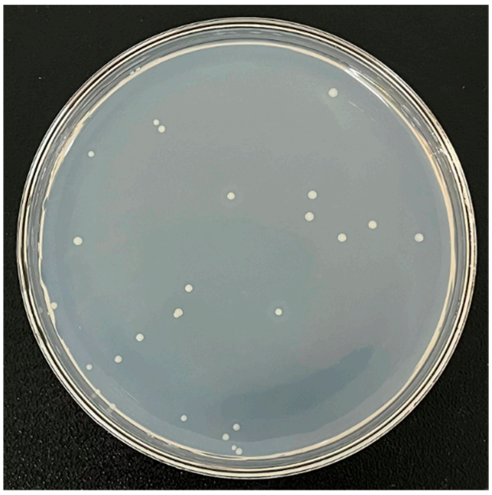

(A)

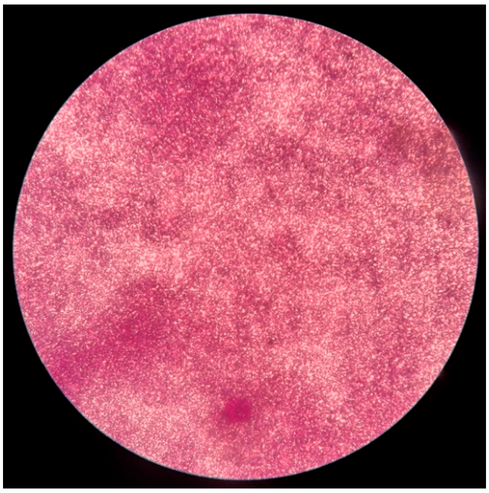

(B)

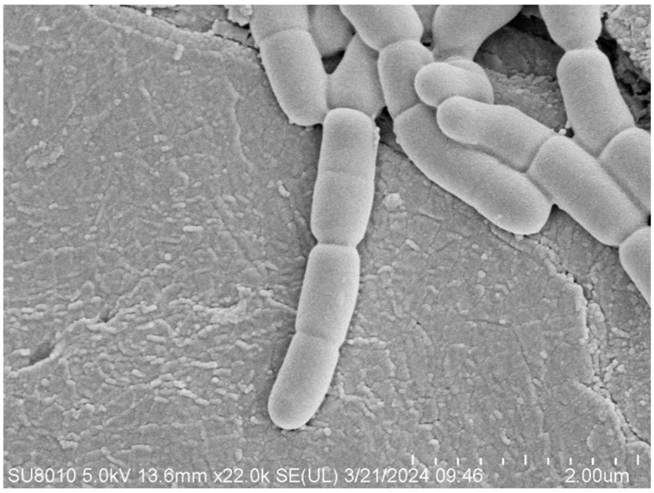

(C)

Fig. S1 (A) Colonial morphology, (B) gram staining result and (C) scanning electron microscopic (SEM) micrograph of the strain PC.

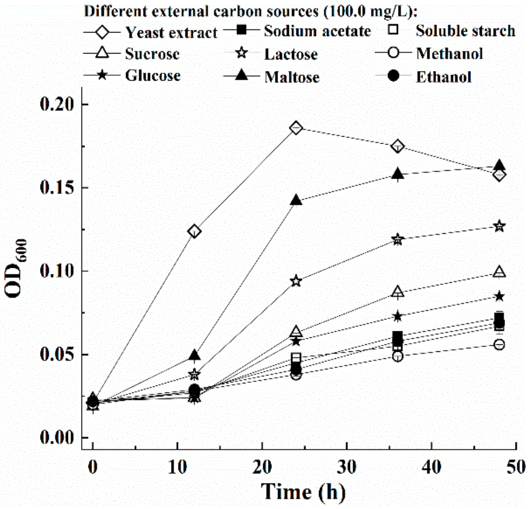
Different external carbon sources (100.0 mg/L):
Yeast extract
Sodium acetate
Soluble starch
Sucrose
Lactose
Methanol
Glucose
Maltose
Ethanol
OD_{600}
Time (h)

(A)

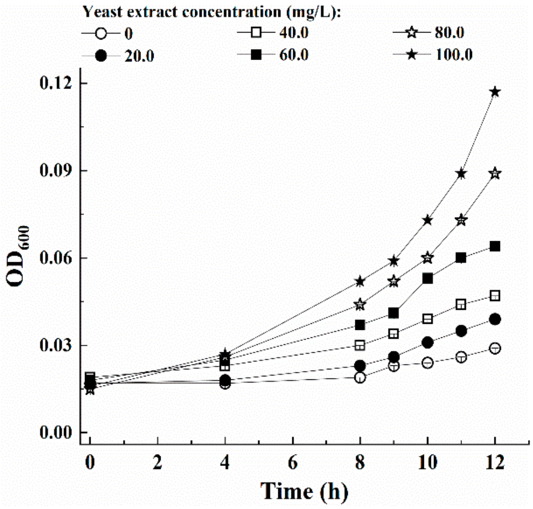
Yeast extract concentration (mg/L):
0
40.0
80.0
20.0
60.0
100.0
OD_{600}
Time (h)

(B)

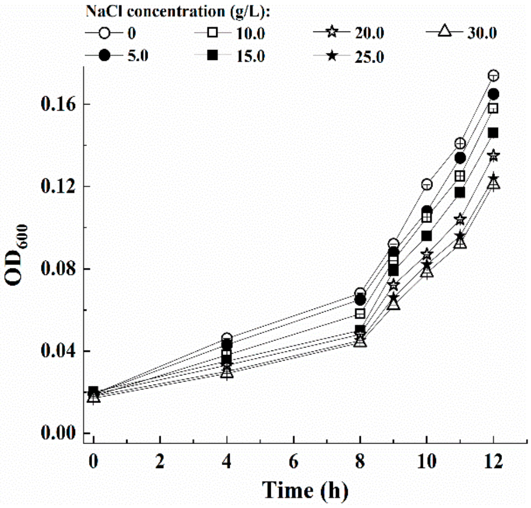
NaCl concentration (g/L):
0
10.0
20.0
30.0
5.0
15.0
25.0
OD_{600}
Time (h)

(C)

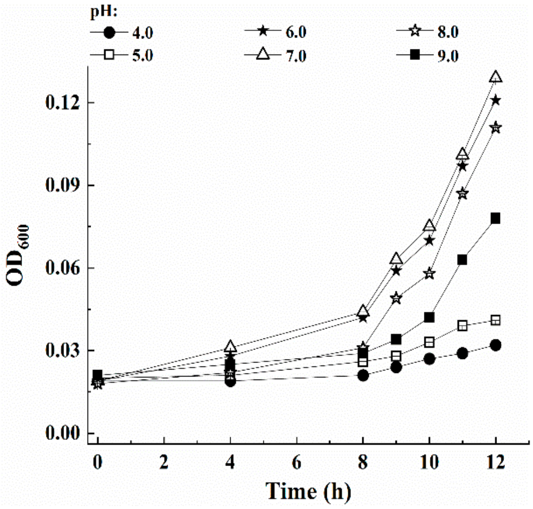
pH:
4.0
6.0
8.0
5.0
7.0
9.0
OD_{600}
Time (h)

(D)

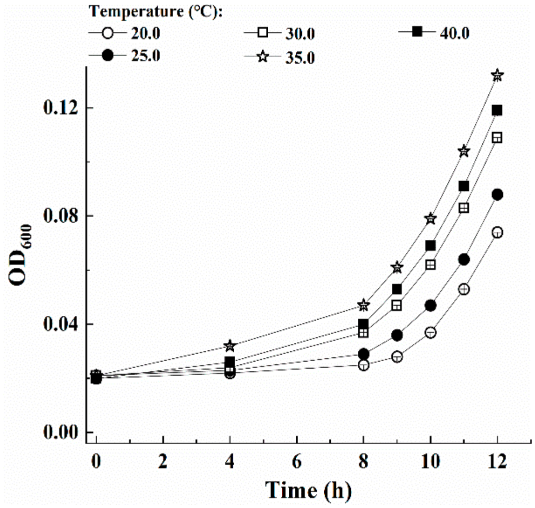
Temperature (°C):
20.0
30.0
40.0
25.0
35.0
OD_{600}
Time (h)

(E)

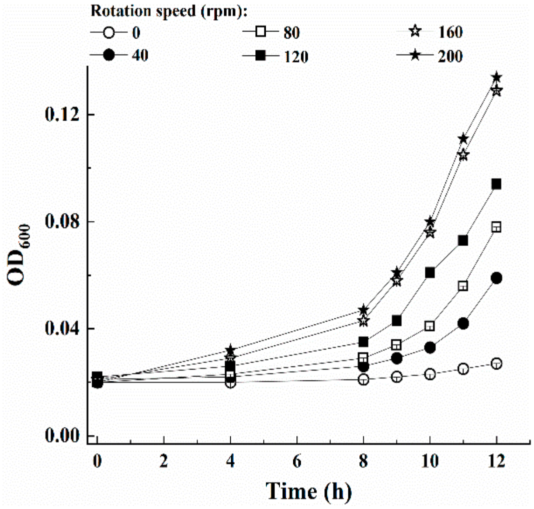
Rotation speed (rpm):
0
80
160
40
120
200
OD_{600}
Time (h)

(F)

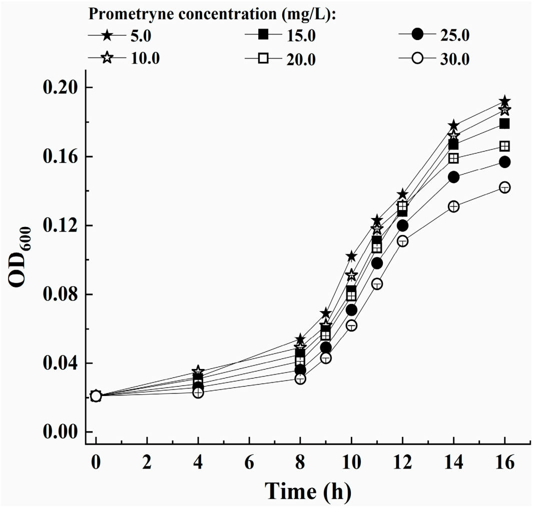

(G)

Fig. S2 Optimization of the conditions for cell growth of the strain PC: (A) type of additional carbon source, (B) yeast extract concentration, (C) NaCl concentration, (D) pH, (E) temperature, (F) rotation speed, and (G) initial prometryne concentration.

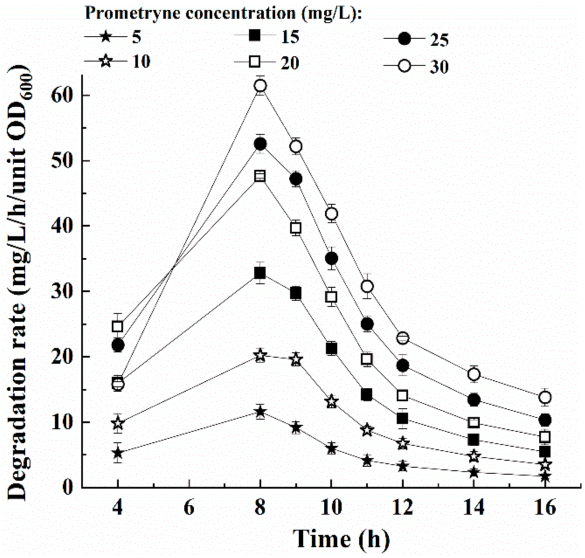

Fig. S3 The average specific degradation rates of prometryne at different initial concentrations.

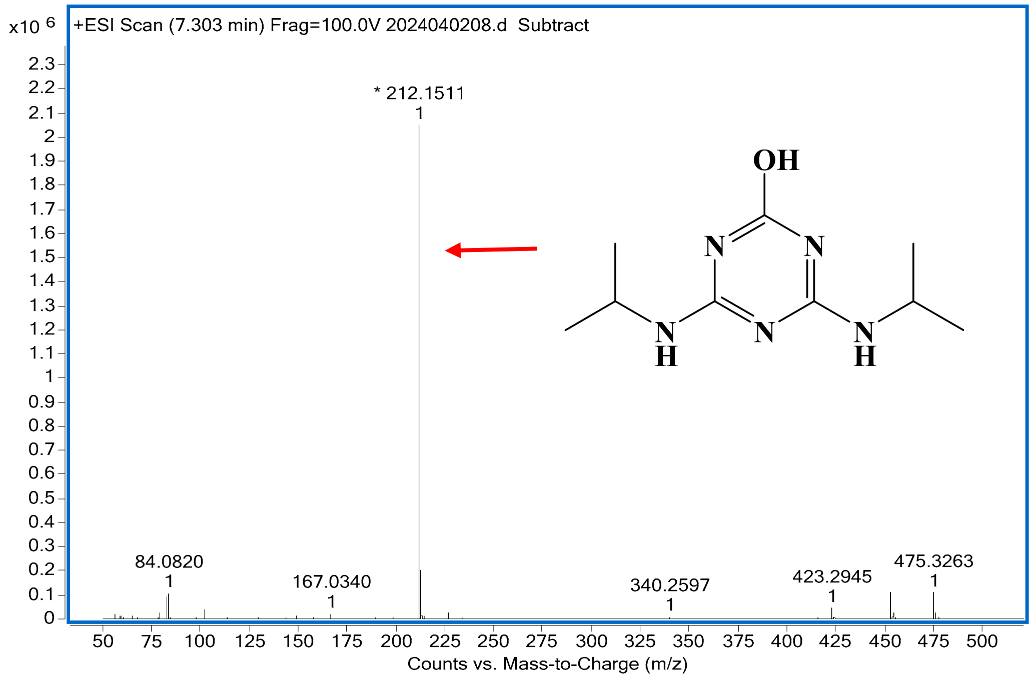
x10 6
+ESI Scan (7.303 min) Frag=100.0V 2024040208.d Subtract
* 212.1511
84.0820
167.0340
340.2597
423.2945
475.3263
OH
N
N
N
N
H
N
H
Counts vs. Mass-to-Charge (m/z)

(A)

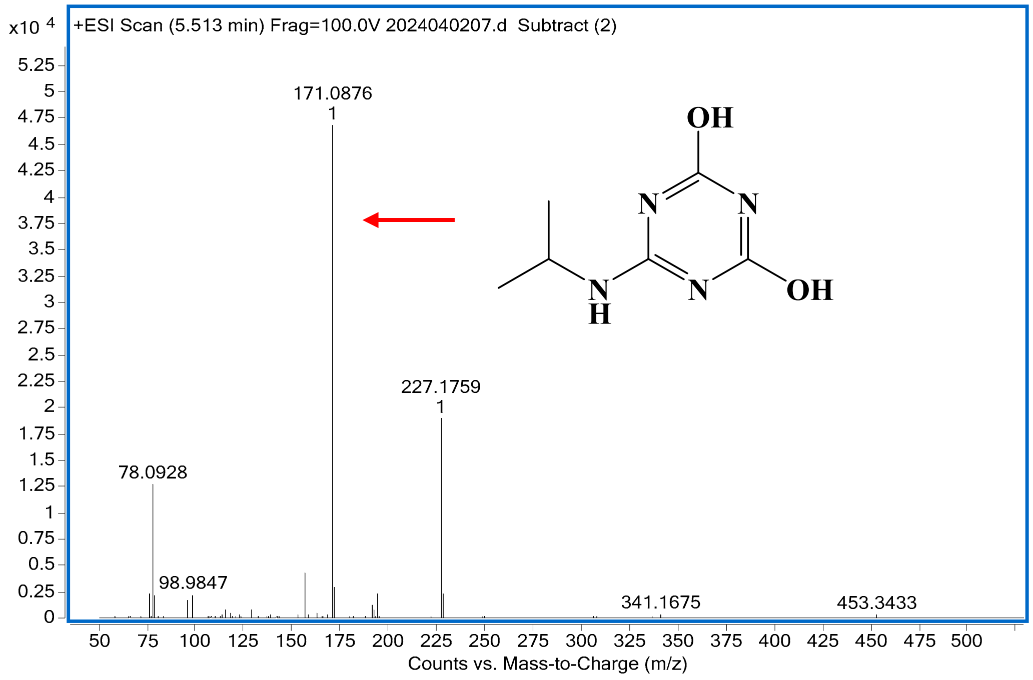
x10 4
+ESI Scan (5.513 min) Frag=100.0V 2024040207.d Subtract (2)
171.0876
227.1759
78.0928
98.9847
341.1675
453.3433
OH
N
N
N
N
H
OH
Counts vs. Mass-to-Charge (m/z)

(B)

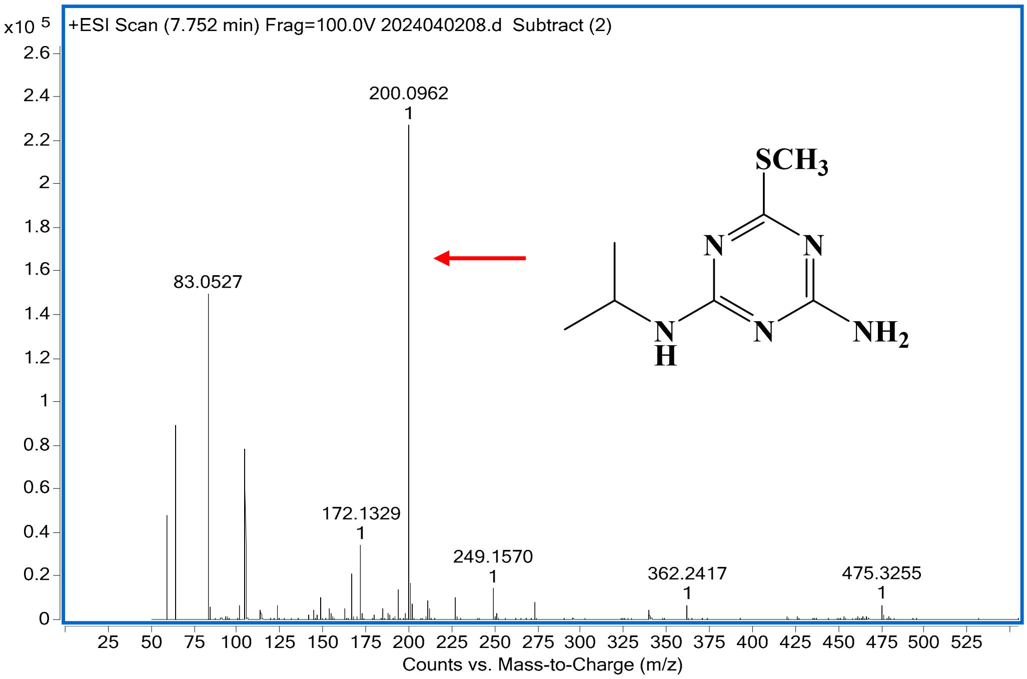

(C)

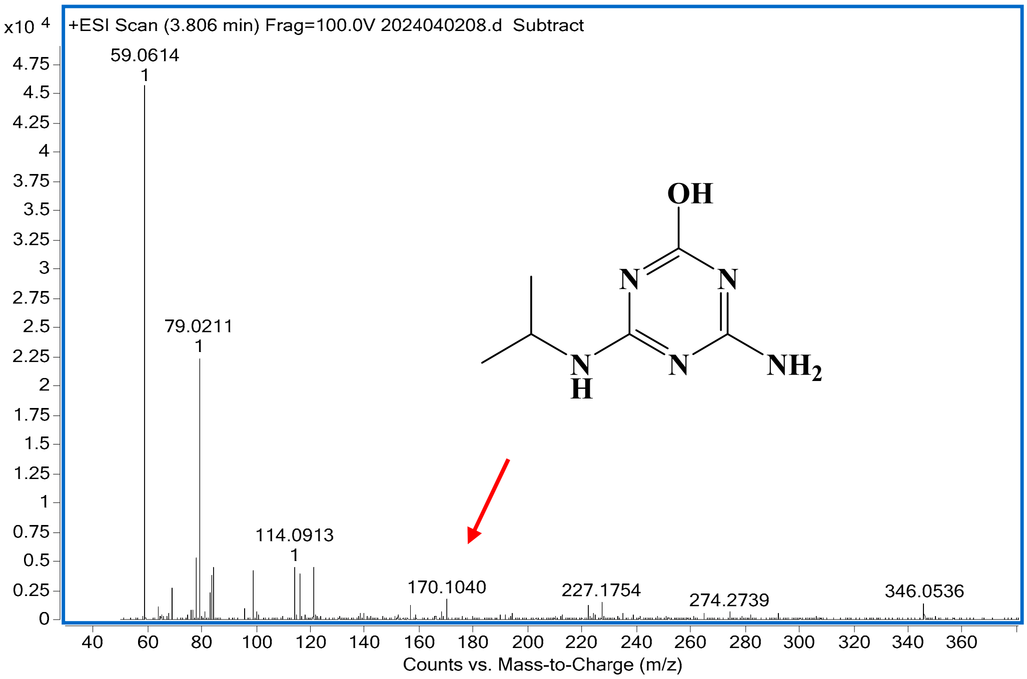

(D)

Fig. S4 TOFMS spectrums of four possible intermediates of prometryne after degradation by the strain PC for 8 h: (A) 4,6-diisopropylamino-1,3,5-triazine-2-ol; (B) 6-isopropylamino-1,3,5-triazine-2,4-diol; (C) N2-isopropyl-6-methylthio-1,3,5-triazine-2,4-diamine; (D) 4-amino-6-isopropylamino-1,3,5-triazine-2-ol.

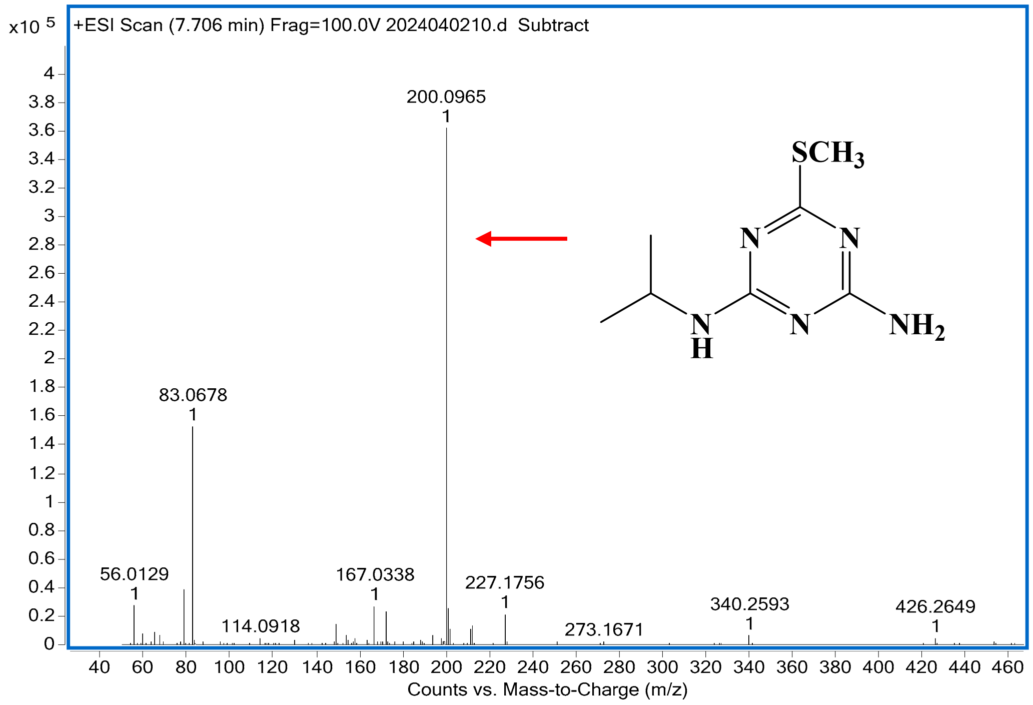

(A)

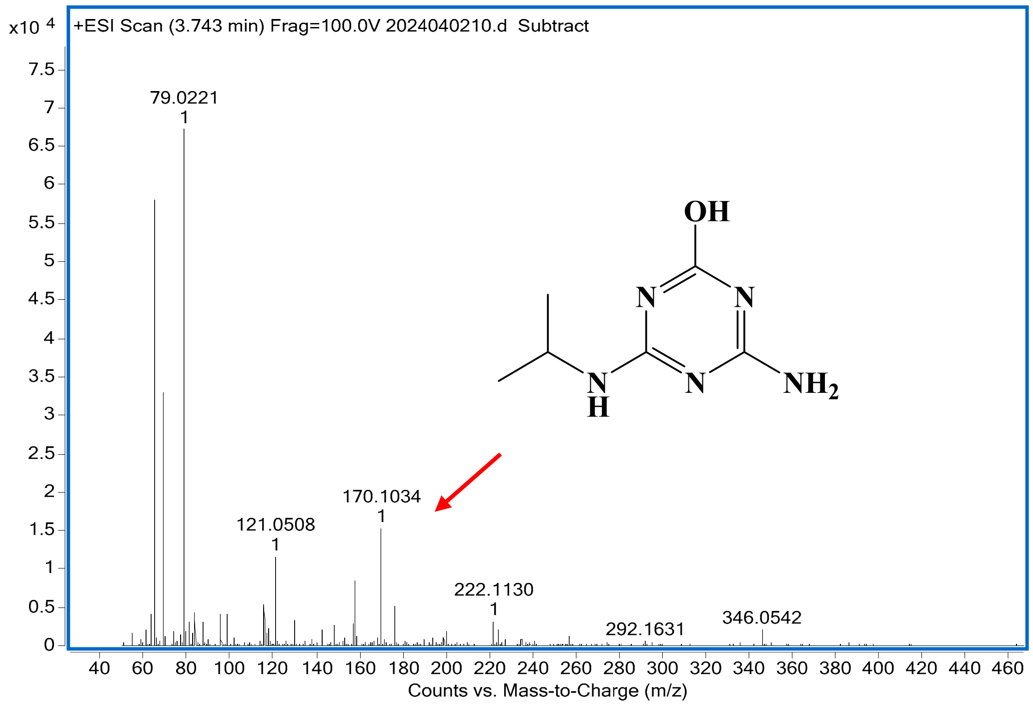

(B)

Fig. S5 TOFMS spectrums of two possible intermediates of prometryne after degradation by the strain PC for 12 h: (A) N2-isopropyl-6-methylthio-1,3,5-triazine-2,4-diamine; (B) 4-amino-6-isopropylamino-1,3,5-triazine-2-ol.

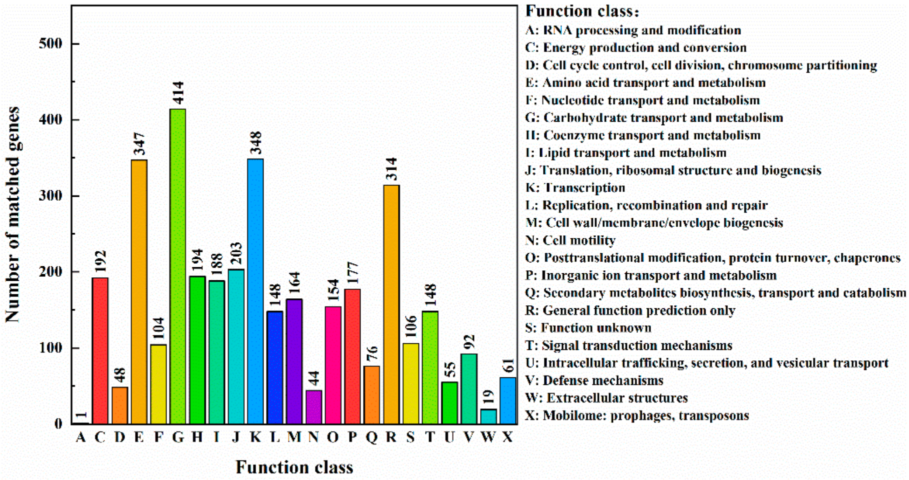

(A)

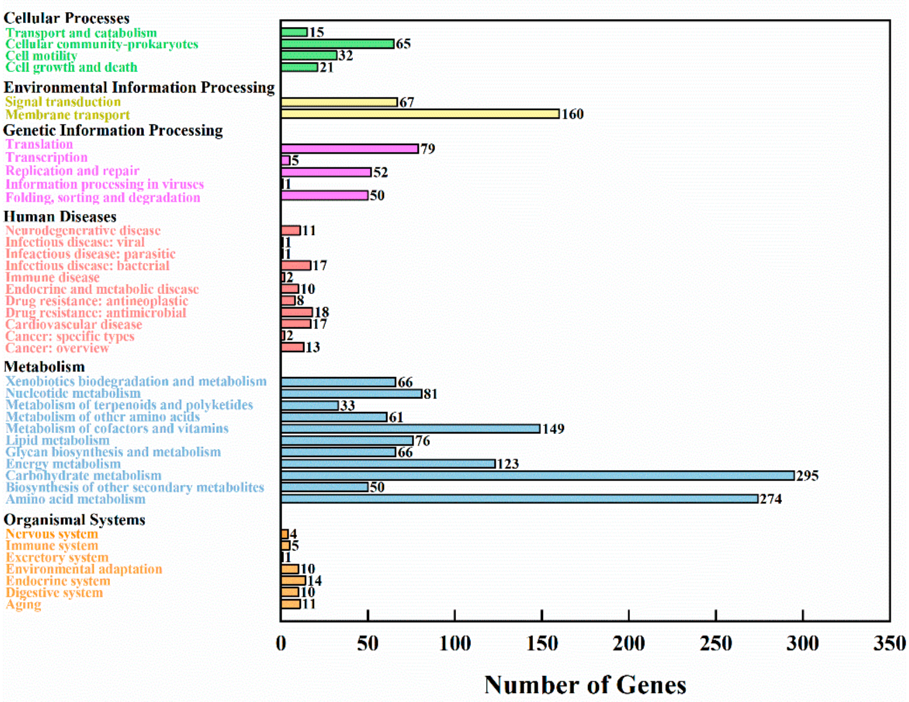

(B)

Fig. S6 The results of gene function annotation of the strain PC based on (A) COG and (B) KEGG databases.

Text S1 Genome sequencing method

1 Genome sequencing

1.1 Total DNA extraction

Total DNA was extracted using a Soil Genomic DNA Extraction Kit (TIANGEN Biotech (Beijing) Co., Ltd.).

1.2 Library construction, quality control and sequencing

Genomic DNA was randomly sheared into short fragments, which were then end-repaired, A-tailed, and ligated with Illumina adapters. The adapter-ligated fragments with adapters were then subjected to size selection, PCR amplification, and purification.

The library was then quantified using both a Qubit 2.0 fluorophotometer and the real-time PCR method, and its size distribution was assessed using a Bioanalyzer. The quantified libraries were pooled and sequenced on Illumina NovaSeq platforms.

2 Genome assembly

2.1 Data processing

The raw data obtained from the process of sequencing (raw data) contained a certain proportion of low-quality data. In order to ensure the accuracy and reliability of the subsequent data analysis results, the original data must be filtered to obtain valid data (clean data).

The specific processing steps were as follows: (1) reads containing low-quality bases (mass value ≤20) above a certain percentage (the default was 40%) were removed; (2) reads with a number of N bases exceeding a certain proportion (the default was 10%) were removed; (3) reads with an overlap with the adapter that exceeded a certain threshold (the default was 15 bp) and had fewer than 3 mismatches were removed.

2.2 Assembly

The specific processing steps for genome assembly with Clean Data were as follows. The assembly was first performed using SOAPdenovo software **[1]**, with different K-mers (the default values were 95,107,119) being selected. According to the project type, the optimal K-mer was chosen,

and other parameters (-d, -u, -R, -F, etc.) were further adjusted. The preliminary assembly result was obtained with the least number of scaffolds. Subsequently, SPAdes software **[2]** was used for assembly, with different K-mers (the default values were 99 and 127) selected based on the project type. The assembly result was obtained with the optimal K-mer and the least number of scaffolds. The Abyss software **[3]** was selected for assembly using K-mer 64. The assembly results from the above three software were integrated using CISA software (https://www.cisa.gov/), and the assembly result with the least scaffolds was selected. The Gapclose software (https://github.com/BGI-Qingdao/TGS-GapCloser) was then employed to fill the gaps in the preliminary assembly results, and lane contamination was removed by filtering out reads with low sequencing depth (less than 0.35 of the average depth), resulting in the final assembly. Finally, fragments shorter than 500 bp were filtered out, and the final result was used for gene prediction.

3 Genome Component prediction

The prediction of genome components encompassed the prediction of coding genes, repetitive sequences, non-coding RNAs, genomic islands, transposons, prophages, and clustered regularly interspaced short palindromic repeat sequences (CRISPR). The specific processing steps were as follows:

The GeneMarkS software **[4]** was used for the prediction of coding genes, while the interspersed repetitive sequences were predicted using the RepeatMasker software (http://www.repeatmasker.org/). The analysis of tandem repeats was conducted using the Tandem Repeats Finder (TRF) software **[5]**, and the prediction of transfer RNA (tRNA) genes was performed using tRNAscan-SE **[6]**. The analysis of ribosomal RNA (rRNA) genes was facilitated by the rRNAmmer software (https://services.healthtech.dtu.dk/services/RNAmmer-1.2/). Small nuclear RNAs (snRNA) were predicted by BLAST against the Rfam database **[7]**, while the IslandPath-DIOMB software **[8]** was used to predict genomic islands, and the PhiSpy software **[9]** was used for prophage prediction. CRISPR identification was performed using the CRISPRdigger software **[10]**.

4 Gene function

A total of six databases were used for the prediction of gene functions, namely Gene Ontology (GO) **[11]**, Kyoto Encyclopedia of Genes and Genomes (KEGG) **[12]**, Clusters of Orthologous Groups (COG) **[13]**, Non-Redundant Protein Database (NR) **[14]**, Transporter Classification Database (TCDB) **[15]** and Swiss-Prot **[16]**. A whole-genome BLAST search was performed against these seven databases, with an E-value cut-off of 10^{-5} and a minimum alignment length percentage of 40%. The secretory proteins were predicted using the SignalP database **[17]**, and Type I-VII proteins secreted by pathogenic bacteria were predicted using EffectiveT3 software **[18]**. Secondary metabolism gene clusters were analyzed using the antiSMASH software **[19]**. For pathogenic bacteria, pathogenicity and drug resistance analyses were performed using the Pathogen Host Interactions (PHI) database **[20]** and the Antibiotic Resistance Genes Database (ARDB) **[21]**. Furthermore, the Comprehensive Antibiotic Resistance Database (CARD) **[22]** was used to analyze antibiotic resistance, while the Carbohydrate-Active Enzymes Database **[23]** was employed to predict Carbohydrate-Active enzymes.

References

[1] Li, R.; Zhu, H.; Ruan, J.; Qian, W.; Fang, X.; Shi, Z.; Li, Y.; Li, S.; Shan, G.; Kristiansen, K.; Li, S.; Yang, H.; Wang, J.; Wang J. De novo assembly of human genomes with massively parallel short read sequencing. *Genome Res*. **2010**, *20*, 265-272.

[2] Bankevich, A.; Nurk, S.; Antipov, D.; Gurevich, A.A.; Dvorkin, M.; Kulikov, A.S.; Lesin, V.M.; Nikolenko, S.I.; Pham, S.; Prjibelski, A.D.; Pyshkin, A.V.; Sirotkin, A.V.; Vyahhi, N.; Tesler, G.; Alekseyev, M.A.; Pevzner, P.A. SPAdes: a new genome assembly algorithm and its applications to single-cell sequencing. *J. Comput. Biol*. **2012**, *19*, 455-477.

[3] Simpson, J.T.; Wong, K.; Jackman, S.D.; Schein, J.E.; Jones, S.J.; Birol, I. ABySS: a parallel assembler for short read sequence data. *Genome Res*. **2009**, *19*, 1117-1123.

[4] Besemer, J.; Lomsadze, A.; Borodovsky, M. GeneMarkS: a self-training method for prediction of gene starts in microbial genomes. Implications for finding sequence motifs in regulatory regions. *Nucleic Acids Res*. **2001**, *29*, 2607-2618.

[5] Benson, G. Tandem repeats finder: a program to analyze DNA sequences. *Nucleic Acids Res*. **1999**, *27*, 573-580.

[6] Chan, P.P.; Lin, B.Y.; Mak, A.J.; Lowe, T.M. tRNAscan-SE 2.0: improved detection and functional classification of transfer RNA genes. *Nucleic Acids Res*. **2021**, *49*, 9077-9096.

[7] Gardner, P.P.; Daub, J.; Tate, J.G.; Nawrocki, E.P.; Kolbe, D.L.; Lindgreen, S.; Wilkinson, A.C.; Finn, R.D.; Griffiths-Jones, S.; Eddy, S.R.; Bateman, A. Rfam: updates to the RNA families database. *Nucleic Acids Res*. **2009**, *37*, D136-D140.

[8] Bertelli, C.; Brinkman, F.S.L. Improved genomic island predictions with IslandPath-DIMOB. *Bioinformatics* **2018**, *34*, 2161-2167.

[9] Akhter, S.; Aziz, R.K.; Edwards, R.A. PhiSpy: a novel algorithm for finding prophages in bacterial genomes that combines similarity- and composition-based strategies. *Nucleic Acids Res*. **2012**, *40*, e126.

[10] Ge, R.; Mai, G.; Wang, P.; Zhou, M.; Luo, Y.; Cai, Y.; Zhou, F. CRISPRdigger: detecting

CRISPRs with better direct repeat annotations. *Sci. Rep.* **2016**, *6*, 32942.

[11] Ashburner, M.; Ball, C.A.; Blake, J.A.; Botstein, D.; Butler, H.; Cherry, J.M.; Davis, A.P.; Dolinski, K.; Dwight, S.S.; Eppig, J.T.; Harris, M.A.; Hill, D.P.; Issel-Tarver, L.; Kasarskis, A.; Lewis, S.; Matese, J.C.; Richardson, J.E.; Ringwald, M.; Rubin, G.M.; Sherlock, G. Gene ontology: tool for the unification of biology. The Gene Ontology Consortium. *Nat. Genet.* **2000**, *25*, 25-29.

[12] Kanehisa, M.; Goto, S.; Hattori, M.; Aoki-Kinoshita, K.F.; Itoh, M.; Kawashima, S.; Katayama, T.; Araki, M.; Hirakawa, M. From genomics to chemical genomics: new developments in KEGG. *Nucleic Acids Res.* **2006**, *34*, D354-D357.

[13] Galperin, M.Y.; Makarova, K.S.; Wolf, Y.I.; Koonin, E.V. Expanded microbial genome coverage and improved protein family annotation in the COG database. *Nucleic Acids Res.* **2015**, *43*, D261-D269.

[14] Li, W.; Jaroszewski, L.; Godzik, A. Tolerating some redundancy significantly speeds up clustering of large protein databases. *Bioinformatics* **2002**, *18*, 77-82.

[15] Saier, M.H. Jr.; Reddy, V.S.; Tamang, D.G.; Västermark, A. The transporter classification database. *Nucleic Acids Res.* **2014**, *42*, D251-D258.

[16] Bairoch, A.; Apweiler, R. The SWISS-PROT protein sequence database and its supplement TrEMBL in 2000. *Nucleic Acids Res.* **2000**, *28*, 45-48.

[17] Petersen, T.N.; Brunak, S.; von Heijne, G.; Nielsen, H. SignalP 4.0: discriminating signal peptides from transmembrane regions. *Nat. Methods* **2011**, *8*, 785-786.

[18] Eichinger, V.; Nussbaumer, T.; Platzer, A.; Jehl, M.A.; Arnold, R.; Rattei, T. EffectiveDB--updates and novel features for a better annotation of bacterial secreted proteins and Type III, IV, VI secretion systems. *Nucleic Acids Res.* **2016**, *44*, D669-D674.

[19] Medema, M.H.; Blin, K.; Cimermancic, P.; de Jager, V.; Zakrzewski, P.; Fischbach, M.A.; Weber, T.; Takano, E.; Breitling, R. antiSMASH: rapid identification, annotation and analysis of secondary metabolite biosynthesis gene clusters in bacterial and fungal genome sequences.

Nucleic Acids Res. **2011**, *39*, W339-W346.

[20] Urban, M.; Pant, R.; Raghunath, A.; Irvine, A.G.; Pedro, H.; Hammond-Kosack, K.E. The Pathogen-Host Interactions database (PHI-base): additions and future developments. *Nucleic Acids Res*. **2015**, *43*, D645-D655.

[21] Liu, B.; Pop, M. ARDB--Antibiotic Resistance Genes Database. *Nucleic Acids Res*. **2009**, *37*, D443-D447.

[22] Jia, B.; Raphenya, A.R.; Alcock, B.; Waglechner, N.; Guo, P.; Tsang, K.K.; Lago, B.A.; Dave, B.M.; Pereira, S.; Sharma, A.N.; Doshi, S.; Courtot, M.; Lo, R.,; Williams, L.E.; Frye, J.G.; Elsayegh, T.; Sardar, D.; Westman, E.L.; Pawlowski, A.C.; Johnson, T.A.; Brinkman, F.S.; Wright, G.D.; McArthur, A.G. CARD 2017: expansion and model-centric curation of the comprehensive antibiotic resistance database. *Nucleic Acids Res*. **2017**, *45*, D566-D573.

[23] Cantarel, B.L.; Coutinho, P.M.; Rancurel, C.; Bernard, T.; Lombard, V.; Henrissat, B. The Carbohydrate-Active EnZymes database (CAZy): an expert resource for Glycogenomics. *Nucleic Acids Res*. **2009**, *37*, D233-D238.
